# Adverse events of herpes zoster vaccination among adults in Saudi Arabia: A cross-sectional study

**DOI:** 10.1371/journal.pone.0360528

**Published:** 2026-10-27

**Authors:** Jehad Aldali, Emadeldin Elsokkary, Ghadah Alnajdi, Marwa Maghrabi, Ruba Subh, Ghala Alakkasi, Sara Alhanshi Yati, Meshal Alzakari

**Affiliations:** 1 Department of Pathology, College of Medicine, Imam Mohammad Ibn Saud Islamic University (IMSIU), Riyadh, Saudi Arabia; 2 Department of Psychology, College of Social Sciences, Imam Mohammad Ibn Saud Islamic University (IMSIU), Riyadh, Saudi Arabia; 3 College of Medicine, Imam Mohammad Ibn Saud Islamic University (IMSIU), Riyadh, Saudi Arabia; Sriwijaya University: Universitas Sriwijaya, INDONESIA

## Abstract

**Background:**

The varicella-zoster virus, which causes herpes zoster upon reactivation, remains a public health concern, particularly among older adults. Although Zostavax and Shingrix are effective vaccines, monitoring adverse events remains important. Limited vaccine uptake and low public awareness in Saudi Arabia further highlight this need. This study seeks to assess the adverse events linked to herpes zoster immunization throughout the Saudi Arabian population.

**Methods:**

This cross-sectional study used a Google Form questionnaire administered from 16 September 2024–15 August 2025.

**Results:**

A total of 382 participants completed the survey. The largest age subgroup was 18–25 years (22.3%). For the first dose, 185 participants (48.4%) did not know the vaccine type, whereas 144 (37.7%) received Zostavax and 53 (13.9%) received Shingrix. Among second-dose recipients (n = 152), 85 participants (55.9%) received Zostavax, 41 (27.0%) received Shingrix, and 26 (17.1%) were uncertain of the vaccine type. The most frequently reported adverse effects after the first dose were fatigue/headache, injection-site pain, and dizziness, whereas chills, injection-site pain, and dizziness were most frequent after the second dose. No adverse effects were reported by 112 participants (29.3%) after the first dose and 28 (18.4%) after the second dose. Participants who received Zostavax reported significantly higher self-reported severity scores than those who received Shingrix after both the first dose (p = 0.003) and the second dose (p < 0.001).

**Conclusion:**

Self-reported symptom severity was higher among participants with chronic disease and varied significantly by age after the first dose. The most commonly reported symptom duration was 1–2 days. No significant gender differences were observed. In unadjusted analyses, participants who reported receiving Zostavax had higher self-reported severity scores than those reporting Shingrix.

## 1. Introduction

Herpes zoster (HZ), commonly known as shingles, is a significant health concern worldwide, resulting from the reactivation of the varicella-zoster virus (VZV), which initially causes chickenpox in children [1–3]. HZ typically presents as a unilateral vesicular rash with a dermatomal distribution, often accompanied by varying degrees of pain described as aching, burning, or tingling [4]. Complications can include postherpetic neuralgia (PHN), bacterial skin infections, and severe neurological and ophthalmological issues such as long-term pain, keratitis, and iridocyclitis [5]. The complications associated with herpes zoster, particularly meningoencephalitis can cause seizures, status epilepticus, cerebral oedema, elevated intracranial pressure, stroke, hydrocephalus, cranial nerve dysfunction, hearing or vision loss, and neurological impairment. Long-term problems include cognitive impairment, memory loss, personality changes, speech and mobility abnormalities, chronic epilepsy, and impaired functional independence. Coma, brain damage, or death might result from severe cases [6]. In addition, the complications associated with herpes zoster, particularly cardiovascular complications, increase the risk of ischaemic stroke, myocardial infarction, coronary artery disease, and other serious cardiovascular events. Reactivating varicella-zoster virus can cause systemic inflammation, endothelial damage, thrombosis, and viral vasculopathy [7].

Globally, HZ incidence has been increasing, particularly among older adults. The incidence rates of HZ are expected to increase significantly after the age of 50, with an estimated 20–30% of the population experiencing the condition during their lifetime. The incidence of HZ in the United States has been steadily increasing, with rates consistently ranging from 3 to 5 per 1,000 person-years across various age groups. The general trend in Saudi Arabia suggests an increasing incidence, which is consistent with global patterns, despite the scarcity of detailed prevalence data. The incidence rates in the Middle East are comparable to those in Europe and North America, with a range of 3–5 per 1,000 person-years [8,9].

Preventive medicine, particularly vaccination, is an essential approach to safeguarding older populations from HZ. Substantial progress over the past decade, researchers have made substantial progress in developing vaccines. At present, two vaccine types are in widespread use: a live-attenuated single-dose vaccine (Zostavax; Merck, Kenilworth, NJ, USA) and a two-dose recombinant adjuvanted glycoprotein E subunit vaccine (Shingrix; GlaxoSmithKline, London, UK) [10,11]. While live-attenuated vaccines have been traditionally recommended, their use in immunocompromised individuals is limited due to the risk of disease from vaccine virus replication. Recombinant subunit vaccines, on the other hand, offer a safer alternative for these individuals by preventing vaccine virus replication [12,13].

Studies worldwide, including in the United States, have demonstrated the safety and efficacy of both vaccines in immunocompetent adults and selected immunocompromised patients. The recombinant HZV vaccine has shown higher efficacy in preventing HZ compared to the live attenuated version, and its nonreplicating nature ensures safety even for immunocompromised individuals [11,14]. In 2008, Chaves reported that the vaccine is safe and effective when administered to healthy individuals in the U.S., with mild adverse effects such as transient rash, fever, and injection site reactions, and rare serious adverse events like transient meningitis [15]. Other common adverse effects include pharyngitis, sore throat, fatigue, and upper respiratory symptoms [15].

Focusing on Saudi Arabia, recent studies have identified significant gaps in knowledge and awareness regarding HZ vaccination among the population aged 50 years and older. A 2023 cross-sectional study by Almumar revealed that 57.2% of participants were aware of the vaccine, with those having primary education levels showing higher acceptance compared to those with higher education. However, only 7.7% of the participants had received the vaccine [16]. Similarly, a study by Allefta found that despite over half of the participants claiming awareness of the HZ vaccine, only a small portion demonstrated high knowledge scores [1].

Therefore, this study aims to evaluate the adverse events associated with herpes zoster vaccination in the Saudi Arabian population.

## 2. Methods

### 2.1. Study design and setting

This cross-sectional study was conducted among adults aged 18–80 years residing in Saudi Arabia who had received the herpes zoster vaccine.

### 2.2. Study participants

The study participants consisted of the overall population residing in Saudi Arabia. The study participants had been administered the herpes zoster vaccine. Participants were recruited based on their accessibility, willingness to participate, and eligibility, specifically adults who reported having received the herpes zoster vaccine. Adverse events data were gathered using a well-organized questionnaire. The adverse were classified according to their severity and the time at which they occurred.

### 2.3. Inclusion and exclusion criteria

**Inclusion Criteria:**

- We selected participants residing in Saudi Arabia who had been administered the herpes zoster vaccine.
- participants who had received at least their initial vaccination or had completed the recommended herpes zoster vaccination.
- Receipt of the vaccine before completing the questionnaire.
- Ability to distinguish the herpes zoster vaccine from the varicella vaccine.
- Ability to understand the study information and questionnaire.
- Provision of electronic informed consent.
- Willingness to participate voluntarily.
- Completion of the mandatory questionnaire sections.

**The exclusion criteria:**

- The exclusion criteria included individuals who had not received the vaccine and those who declined to participate in this study.
- Individuals residing outside Saudi Arabia. Individuals who declined or withdrew informed consent.
- Questionnaire responses submitted by another person on the participant’s behalf.
- Incomplete questionnaires or responses with substantial missing data.
- Duplicate questionnaire submissions.

### 2.4. Sample size calculation

The study’s sample size was calculated using the ‘Raosoft’ power calculator [17]. According to this calculation, a sample size of 382 participants was deemed sufficient to achieve a 95% confidence level with a 5% margin of error. A total of 382 participants completed the survey, meeting the required sample size.

### 2.5. Ethical declaration

The Institutional Review Board, College of Medicine, Imam Muhammad bin Saud Islamic University approved the study on 9 January 2024 (Ref. 584–2023), performed in accordance with the relevant guidelines and regulations.

### 2.6. Study questionnaire development

To assess adverse events associated with herpes zoster vaccination in Saudi Arabia, we developed a researcher-designed electronic self-administered questionnaire using Google Forms. The survey was distributed via social media platforms (email, Twitter, Instagram, and WhatsApp) to adults aged 18–80 years who had received the herpes zoster vaccine between 9 January 2024 and 15 August 2025. The questionnaire items were generated based on a review of the existing literature on herpes zoster vaccination, as well as input from the research team and key stakeholders.

The questionnaire was adapted from a previous study to meet the needs of this research [18]. The draft questionnaire was reviewed by a principal investigator with expertise in vaccine research. The principal investigator provided feedback on the content, wording, and structure of the questions. Based on his input, the questionnaire was refined and revised.

The revised questionnaire was then pilot-tested among 20 individuals representative of the target study population to evaluate clarity, comprehension, and relevance. Participants were asked to provide feedback on the questionnaire content, and minor wording modifications were made based on pilot feedback to enhance question clarity.

The final questionnaire consisted of the following sections: demographic information (e.g., age, gender, smoking status, and current health status), vaccination-related information (number of doses received, history of shingles before vaccination, and vaccine type), and post-vaccination adverse events. For each vaccine dose, participants were asked to report whether they experienced any adverse effects, indicate the onset and duration of symptoms, report whether they used medications to reduce symptom severity, and self-rate the overall severity of post-vaccination adverse effects using four predefined response categories: no symptoms, mild, moderate, or severe. Participants provided informed consent prior to answering the questionnaire. Comprehensive information about the study was provided to ensure their understanding and to enable them to voluntarily and thoughtfully decide whether to participate. They were informed that participation was voluntary and that they could withdraw from the study at any time without providing a reason or facing any consequences. Only those who selected “Yes” to the informed consent question were allowed to proceed with the survey.

The final questionnaire was approximately 10 minutes in length. All items were formatted as multiple-choice responses to facilitate data collection and analysis. To minimize misclassification, the questionnaire clearly specified the herpes zoster (shingles) vaccine and distinguished it from other vaccines such as the varicella (chickenpox) vaccine. Participants were also asked about relevant clinical contexts, including prior shingles infection and underlying health conditions. However, as the data were self-reported and not verified through medical records, the possibility of misclassification bias cannot be excluded.

### 2.7. Statistical analysis

The severity of adverse effects was assessed using a researcher-developed self-reported ordinal item for each vaccine dose. Participants rated overall post-vaccination severity using four predefined response categories: 0 = no symptoms, 1 = mild, 2 = moderate, and 3 = severe. The severity ratings represented participants’ global self-assessment of symptom burden and were not assigned by clinicians. Data were analyzed using IBM SPSS Statistics version 28. Descriptive statistics were reported as frequencies and percentages. Because the primary severity outcome was ordinal, non-parametric methods were used for bivariate comparisons. The Mann–Whitney U test was used to compare severity scores between two independent groups, including gender, smoking status, chronic disease status, and reported vaccine type. The Kruskal–Wallis test was used to compare severity across the predefined age groups. When the Kruskal–Wallis test was statistically significant, Dunn’s pairwise post-hoc test with Bonferroni adjustment was performed.

For participants who received both vaccine doses (n = 152), paired analyses were conducted to compare outcomes after the first and second doses. McNemar’s test was used for paired binary outcomes, including individual adverse effects and medication use. The Stuart–Maxwell test was used to compare the paired multi-category symptom-onset distributions, while the Wilcoxon signed-rank test was used to compare ordinal severity and symptom duration between doses. Holm adjustment was applied to the symptom-specific McNemar comparisons to account for multiple testing.

An overall adverse-event count was also calculated for each participant by summing the specific adverse-effect symptoms reported across all available dose-specific checklists, providing a descriptive measure of overall adverse-event burden regardless of dose.

Effect sizes were calculated to aid interpretation. For Mann–Whitney U and Wilcoxon signed-rank tests, effect size r was calculated as |Z|/√N. For the Kruskal–Wallis test, epsilon-squared (ε^2^) was calculated as (H − k + 1)/(n − k), where H is the Kruskal–Wallis statistic, k is the number of groups, and n is the total sample size.

To identify factors independently associated with greater self-reported severity after the first dose, a multivariable ordinal logistic regression model was fitted using the full first-dose sample (N = 382). The model included gender, age (≤50 vs. > 50 years), smoking status, chronic disease status, immune-related disease status, previous herpes zoster diagnosis, and reported vaccine type. Because age was originally collected in grouped categories, including 41–50 years, an exact <50 versus ≥50 classification could not be reconstructed; therefore, ≤ 50 versus >50 years was used for the multivariable analysis. Vaccine type was entered as a categorical variable, including participants who were unable to identify the vaccine received. Adjusted odds ratios (aORs) with 95% confidence intervals (CIs) were reported. The proportional-odds assumption and multicollinearity among predictors were assessed before interpreting the model. All statistical tests were two-sided, and p < 0.05 was considered statistically significant.

## 3. Results

A total of 382 participants completed the survey. The sample was approximately balanced by gender and was predominantly Saudi (95.5%, n = 365), a majority were married (62.8%, n = 240) and non-smoking (82.2%, n = 314). The largest age subgroup was 18–25 years (22.3%), and 31.9% reported a chronic disease. Most participants had received one vaccine dose (60.2%). Vaccine type was unknown to 48.4% of participants after the first dose and to 17.1% of second-dose recipients. Full participant characteristics and vaccination history are presented in Table 1.

**Table 1:** Participant characteristics, health status, and vaccination history.

Variables	Frequency	Percent (%)
**Gender**		
Male	189	49.5%
Female	193	50.5%
**Marital status**		
Married	240	62.8%
Single	142	37.2%
**Age groups (years)**		
18-25	85	22.3%
26-30	68	17.8%
31-40	76	19.9%
41-50	66	17.3%
51-60	57	14.9%
>60	30	7.9%
**Nationality**		
Saudi	365	95.5%
Non-Saudi	17	4.5%
**Smoking History**		
Non-smokers	314	82.2%
Smokers	68	17.8%
**Chronic disease status**		
No chronic disease	260	68.1%
With chronic disease	122	31.9%
**Immune-related disease status**		
No immune-related disease	363	95.0%
With immune-related disease	19	5.0%
**Number of vaccine doses received**		
One dose	230	60.2%
Two doses	152	39.8%
**History of shingles before first dose**		
No	249	65.2%
Yes	133	34.8%
**First-dose vaccine type**		
Zostavax	144	37.7%
Shingrix	53	13.9%
Don’t know	185	48.4%
**Among second-dose recipients only (n = 152)**		
**History of shingles before second dose**		
No	51	33.6%
Yes	101	66.4%
**Second-dose vaccine type**		
Zostavax	85	55.9%
Shingrix	41	27.0%
Don’t know	26	17.1%

***Note.*** Percentages for variables under “Among second-dose recipients only” were calculated using the subgroup of participants who received a second dose (n = 152).

Table 2 summarizes adverse effects, symptom onset, self-reported severity, symptom duration, and medication use after vaccination. Descriptive results for the first dose are presented for the full sample (N = 382). To permit valid within-participant comparisons between doses, paired analyses were restricted to the 152 participants who received both doses. Participants adverse events—such as fatigue, headache, and injection-site pain remained stable between the first and second doses, with no participant symptom frequency showing a statistically significant change (all p > 0.05). While fever and chills showed a descriptive increase following the second dose (23.7% vs. 13.8%), but did not achieve statistical significance within the paired analysis (p = 0.403). Self-reported severity was modestly lower after the second dose than after the first dose (Wilcoxon signed-rank test: Z = −2.973, p = 0.003, r = 0.241). the duration of adverse effects shortened significantly (p < 0.001), with 50.0% of participants resolving symptoms within 1–2 days after the second dose compared to 34.9% after the first. In contrast, symptom-onset distributions did not differ significantly between doses (Stuart–Maxwell p = 0.379), and medication use was similar after the first and second doses (70.4% vs. 69.1%; McNemar p = 0.804). Although some individual adverse effects differed descriptively between doses, none remained statistically significant after Holm adjustment for multiple symptom-specific comparisons.

**Table 2:** Adverse effects and symptom onset, self-reported severity, duration, and medication use after herpes zoster vaccination.

Parameter	First Dose, All Participants N = 382, n (%)	First Dose Among Two-Dose Recipients n = 152, n (%)	Second Dose n = 152, n (%)	Paired p-value
**Adverse effects**				
Fatigue/headache	82 (21.5)	26 (17.1)	18 (11.8)	1.000
Injection-site pain	68 (17.8)	31 (20.4)	32 (21.1)	1.000
Muscle/joint pain or feeling unwell	23 (6.0)	7 (4.6)	12 (7.9)	1.000
Fever/chills	49 (12.8)	21 (13.8)	36 (23.7)	0.403
Dizziness	58 (15.2)	33 (21.7)	21 (13.8)	1.000
Diarrhea	28 (7.3)	13 (8.6)	10 (6.6)	1.000
Loss of concentration	32 (8.4)	17 (11.2)	10 (6.6)	1.000
Change in blood glucose level	22 (5.8)	15 (9.9)	15 (9.9)	1.000
Vomiting	22 (5.8)	12 (7.9)	6 (3.9)	1.000
Skin itching/rash	23 (6.0)	17 (11.2)	7 (4.6)	0.840
Swollen lymph nodes	18 (4.7)	16 (10.5)	10 (6.6)	1.000
Abdominal cramps	13 (3.4)	7 (4.6)	4 (2.6)	1.000
Rapid heartbeat	11 (2.9)	8 (5.3)	7 (4.6)	1.000
Respiratory tract infection	10 (2.6)	8 (5.3)	4 (2.6)	1.000
Chest pain	8 (2.1)	6 (3.9)	6 (3.9)	1.000
Swollen lips	3 (0.8)	1 (0.7)	0 (0.0)	1.000
Menstrual disorder	1 (0.3)	1 (0.7)	3 (2.0)	1.000
No adverse effects	112 (29.3)	18 (11.8)	28 (18.4)	0.114
**Symptom onset**				**0.379**
No symptoms	121 (31.7)	23 (15.1)	30 (19.7)	
First day	87 (22.8)	39 (25.7)	39 (25.7)	
Second day	129 (33.8)	71 (46.7)	67 (44.1)	
Third day	45 (11.8)	19 (12.5)	16 (10.5)	
**Self-reported severity**				0.003*
No symptoms	115 (30.1)	22 (14.5)	28 (18.4)	
Mild	41 (10.7)	10 (6.6)	15 (9.9)	
Moderate	165 (43.2)	83 (54.6)	84 (55.3)	
Severe	61 (16.0)	37 (24.3)	25 (16.4)	
**Duration of adverse effects**				<0.001*
No symptoms	116 (30.4)	22 (14.5)	30 (19.7)	
1–2 days	153 (40.1)	53 (34.9)	76 (50.0)	
3 days	93 (24.3)	66 (43.4)	44 (28.9)	
4 days or more	20 (5.2)	11 (7.2)	2 (1.3)	
**Medication use**				**0.804**
Took medication to reduce severity	152 (39.8)	107 (70.4)	105 (69.1)	
Did not take medication	230 (60.2)	45 (29.6)	47 (30.9)	
				

***Note.*** First-dose descriptive percentages in the first column are based on all participants (N = 382). Paired comparisons are based exclusively on participants who received both doses (n = 152). Individual adverse-effect comparisons were performed using exact McNemar tests with Holm adjustment for multiple comparisons; the displayed p-values for these rows are Holm-adjusted. Symptom-onset distributions were compared using the Stuart–Maxwell test. Self-reported severity and symptom duration were compared using the Wilcoxon signed-rank test, and medication use was compared using McNemar’s test. *p < 0.05. “No adverse effects” and the “No symptoms” responses under onset, severity, and duration were obtained from separate questionnaire items and therefore may have slightly different frequencies.

Across both dose-specific adverse-effect checklists, a total of 672 individual symptom reports were recorded, comprising 471 after the first dose and 201 after the second dose. Overall, 272 participants (71.2%) reported at least one specific adverse-effect symptom across one or both doses. The median number of reported symptoms per participant across the available doses was 1 (IQR: 0–3).

### 3.1. Self-reported severity of adverse effects according to participant characteristics

Table 3 presents bivariate comparisons of self-reported adverse-effect severity to gender, reported vaccine type, smoking status, and chronic disease status. No statistically significant differences were observed according to gender (p = 0.390, r = 0.044) and second doses (p = 0.275, r = 0.089) or smoking status after either dose. In the unadjusted analyses, participants who reported receiving Zostavax had higher severity scores than those who reported receiving Shingrix after the first dose (p = 0.003, r = 0.213) and the second dose (p < 0.001, r = 0.474). Participants with chronic disease also reported higher severity scores after both the f]irst dose (p < 0.001, r = 0.258) and the second dose (p = 0.011, r = 0.205). Potential predictors of first-dose severity were subsequently evaluated in the multivariable analysis.

**Table 3:** Bivariate comparisons of self-reported severity of adverse effects according to participant characteristics.

Characteristic	Dose	Groups, n (Mean Rank)	Mann–Whitney U	Z	p-value	Effect size (r)
**Gender**	First	Male, 189 (186.88); Female, 193 (196.03)	17365.0	−0.860	0.390	0.044
	Second	Male, 72 (72.78); Female, 80 (79.85)	2612.0	−1.092	0.275	0.089
**Reported vaccine type**	First	Zostavax, 144 (105.83); Shingrix, 53 (80.44)	2832.5	−2.990	0.003*	0.213
	Second	Zostavax, 85 (74.11); Shingrix, 41 (41.50)	840.5	−5.320	<0.001*	0.474
**Smoking status**	First	Non-smoker, 314 (195.86); Smoker, 68 (171.38)	9308.0	−1.760	0.078	0.090
	Second	Non-smoker, 133 (78.47); Smoker, 19 (62.74)	1002.0	−1.609	0.108	0.131
**Chronic disease status**	First	No, 260 (173.15); Yes, 122 (230.61)	11089.0	−5.035	<0.001*	0.258
	Second	No, 95 (70.16); Yes, 57 (87.07)	2105.0	−2.533	0.011*	0.205

***Note.*** Comparisons were performed using the Mann–Whitney U test. Participants who reported that they did not know the vaccine type were excluded only from the vaccine-type comparisons. *p < 0.05.

### 3.2. Self-reported severity of adverse effects according to age

Table 4 shows a statistically significant difference in self-reported severity across age groups after the first dose (H = 29.895, p < 0.001, ε² = 0.066). Dunn–Bonferroni post-hoc comparisons showed that participants aged 18–25 years reported significantly lower severity than those aged 31–40 years (adjusted p < 0.001), 41–50 years (adjusted p = 0.004), and >60 years (adjusted p = 0.001). No other pairwise comparisons were statistically significant. After the second dose, severity did not differ significantly across age groups (H = 10.100, p = 0.072, ε² = 0.035); therefore, no post-hoc comparisons were performed.

**Table 4:** Self-reported severity of adverse effects t across age group.

Dose	Age	N	Mean Rank	Kruskal-Wallis H	df	p-value	Effect size (ε²)
First dose	18–25	85	145.42	29.895	5	<0.001*	0.066
	26–30	68	190.26				
	31–40	76	221.18				
	41–50	66	207.52				
	51–60	57	181.44				
	>60	30	233.53				
Second dose	18–25	19	59.61	10.100	5	0.072	0.035
	26–30	27	83.61				
	31–40	50	84.25				
	41–50	37	77.47				
	51–60	14	55.43				
	>60	5	76.60				

***Note***. Overall age-group comparisons were performed using the Kruskal–Wallis test. Following the significant first-dose test, Dunn’s post-hoc comparisons with Bonferroni adjustment identified significant differences between 18–25 and 31–40 years (adjusted p < 0.001), 18–25 and 41–50 years (adjusted p = 0.004), and 18–25 and >60 years (adjusted p = 0.001). No other pairwise comparisons were significant. No post-hoc analysis was conducted for the second dose because the overall Kruskal–Wallis test was not statistically significant. *p < 0.05.

### 3.3. Multivariable analysis of self-reported severity after the first dose

A multivariable ordinal logistic regression analysis was performed to identify factors independently associated with higher self-reported severity after the first dose (N = 382). The overall model was statistically significant (χ^2^(8) = 106.24, p < 0.001; Nagelkerke R^2^ = 0.264), and the proportional-odds assumption was not violated (p = 0.203).

As shown in Table 5, a previous diagnosis of herpes zoster was independently associated with greater severity (aOR = 4.62, 95% CI: 2.84–7.52, p < 0.001), as was chronic disease status (aOR = 2.39, 95% CI: 1.49–3.85, p < 0.001). Participants aged >50 years also had higher odds of reporting greater severity than those aged ≤50 years (aOR = 1.72, 95% CI: 1.00–2.96, p = 0.049). In contrast, gender, smoking status, and immune-related disease status were not independently associated with severity.

**Table 5:** Factors associated with self-reported severity after the first dose.

Variable	aOR	95% CI	p-value
Female vs. male	0.91	0.60–1.40	0.682
Age > 50 vs. ≤ 50 years	1.72	1.00–2.96	0.049*
Smoker vs. non-smoker	0.96	0.56–1.66	0.895
Chronic disease: yes vs. no	2.39	1.49–3.85	<0.001*
Immune-related disease: yes vs. no	0.83	0.32–2.15	0.702
Previous herpes zoster diagnosis: yes vs. no	4.62	2.84–7.52	<0.001*
Reported Zostavax vs. Shingrix	1.34	0.72–2.48	0.350
Vaccine type unknown vs. Shingrix	0.56	0.31–1.01	0.054

***Note.*** aOR = adjusted odds ratio; CI = confidence interval. The dependent variable was self-reported severity coded as 0 = no symptoms, 1 = mild, 2 = moderate, and 3 = severe. Reported Shingrix was the reference category for vaccine type. *p < 0.05.

Importantly, the unadjusted difference previously observed between participants reporting Zostavax and Shingrix was no longer statistically significant after adjustment for the other variables in the model (aOR = 1.34, 95% CI: 0.72–2.48, p = 0.350).

## 4. Discussion

Herpes zoster (HZ), a reactivation of the varicella-zoster virus, remains a significant public health concern, particularly among older adults [1]. Although vaccines such as Zostavax and Shingrix have demonstrated high efficacy, the occurrence of post-vaccination adverse events continues to warrant attention [15]. In Saudi Arabia, both public awareness and vaccine uptake remain limited [16]. Given these concerns, this study aimed to evaluate the adverse events associated with herpes zoster vaccination in the Saudi Arabian population.

The demographic and health characteristics of the participants in this study provide important context for interpreting vaccination patterns and post-vaccination outcomes. Although herpes zoster is more common in adults aged 50 and older, our study included a younger population. This may reflect local vaccination practices which younger participants receive the vaccine because of previous infection or underlying chronic or immune-related conditions. However, this also raises the possibility of misclassification, as some participants may have confused the herpes zoster vaccine with other vaccines such as varicella. Although the questionnaire aimed to distinguish between these vaccines, reliance on self-reported data remains a limitation. Although herpes zoster is more common among adults aged 50 years and older, our study included a relatively younger population. This may reflect local vaccination practices in which younger individuals receive the vaccine because of previous infection or underlying chronic or immune-related conditions [19]. However, it also raises the possibility of misclassification, as some participants may have confused the herpes zoster vaccine with the varicella vaccine [20]. Although the questionnaire clearly distinguished between the two vaccines, reliance on self-reported data remains a limitation. The inclusion of younger participants may limit the generalizability of the findings to older adults but provides insight into post-vaccination responses in a less-studied group. The study population had a balanced gender distribution and consisted predominantly of Saudi nationals. Chronic diseases were reported more frequently than immune-related conditions. Although all participants indicated that they had completed the recommended vaccination schedule, a considerable proportion could not identify the specific herpes zoster vaccine they had received. This uncertainty highlights potential gaps in vaccine literacy and communication between healthcare providers and vaccine recipients, consistent with findings previously reported by Hurley *et al*. (2018) [20]. A considerable proportion of participants also reported having experienced herpes zoster before vaccination, suggesting that previous infection may have motivated their decision to receive the vaccine [15,21].

Among participants who received both doses, paired analyses showed modestly lower overall self-reported severity and shorter symptom duration after the second dose. Although some individual symptoms differed descriptively between doses, none remained statistically significant after adjustment for multiple comparisons. Moderate symptoms were the most commonly reported severity category, and symptoms most frequently lasted 1–2 days, consistent with typical post-vaccination response timelines. Medication use was similar after the first and second doses among participants who received both doses.. These findings are consistent with previous studies reporting similar local and systemic reactions [14,22]. However, the present study additionally identified increased medication use following the second dose, a behavioral response that has received limited attention in earlier research [23].

No statistically significant differences in self-reported adverse-effect severity were observed between male and female participants after either dose. This finding is consistent with previous research reporting broadly comparable vaccine safety profiles across genders [14,15].

Furthermore, we observed statistically significant differences in severity of adverse effects between the two vaccine types. Participants who received Zostavax experienced more intense symptoms than those who received Shingrix. This finding aligns with prior research indicating that live attenuated vaccines like Zostavax may trigger a stronger immune response, especially in vulnerable participants, due to the replication of the attenuated virus [11]. On the other hand, Shingrix, a non-live recombinant vaccine enhanced with the AS01B adjuvant, is known to produce strong immune responses with a lower risk of severe adverse effects [13].

Several factors may explain the unadjusted association observed between vaccine type and symptom severity.]. First, vaccine-type misclassification is possible, particularly because a substantial proportion of participants were unable to identify the vaccine they had received. Second, the study relied on retrospective self-reported data, making differential recall of both vaccine type and adverse effects possible. Third, the non-probability sampling approach may have introduced selection bias. Finally, participant characteristics associated with symptom severity, such as age and chronic disease status, may have differed across vaccine groups. Therefore, the comparison between vaccine types in this study should be considered exploratory rather than confirmatory.

Our study also observed that non-smokers tended to report slightly more noticeable adverse effects than smokers, although the difference was not statistically significant. Therefore. this finding should be interpreted cautiously and does not establish an association between smoking status and post-vaccination adverse effects.

In the multivariable analysis, previous herpes zoster diagnosis and chronic disease remained independently associated with greater self-reported severity, while the association with age was more modest. Previous herpes zoster research suggests that individuals with chronic conditions, including cardiovascular and pulmonary diseases, may experience greater reactogenicity because of baseline inflammation and immune dysregulation [24]. The modest age-related association may also reflect immunosenescence and the increased prevalence of comorbidities among older adults, although newer vaccine formulations remain effective across age groups [22]. These findings highlight the complex relationship between age, chronic disease, previous herpes zoster, and post-vaccination symptom severity [3]. These findings support tailored pre-vaccination counselling to manage expectations and address the needs of potentially vulnerable populations.

### 4.1. Limitation

The non-probability online sampling approach and the composition of the study sample may limit the generalizability of the findings. The small sample size, resulting from the online distribution of a self-administered questionnaire, may limit the generalizability of the findings. As the study design was cross-sectional and relied on self-reported data, recall bias is possible, particularly because the survey may have been completed sometime after vaccination. In addition, a large proportion of participants (17.1%) were unable to recall the specific vaccine brand they had received. This may have affected the accuracy of the reported onset, duration, and severity of adverse effects. Furthermore, prior beliefs or misconceptions about vaccines may have influenced participants’ responses.

As most participants were aged 18–40 years, this may limit the generalizability of our findings to older adults, who are at higher risk of herpes zoster. This sampling pattern may also affect the interpretation of reported adverse effects, as younger individuals may respond differently to vaccination.

One of the limitations of this study is the potential underrepresentation of older adults in the sample, as many individuals aged 60 and above may be hesitant to receive the herpes zoster vaccine due to concerns about their weakened immune systems. With aging, the immune system undergoes significant changes, including a decline in innate immune responses and the development of a chronic proinflammatory state known as “inflammaging.”

Symptom severity was assessed using a self-reported ordinal item in the researcher-developed questionnaire rather than a clinician-verified grading system. Therefore, the severity categories reflect participant-perceived symptom burden and may be subject to individual differences in interpretation, symptom tolerance, and recall. Although this approach was practical for a large-scale online survey, it may have introduced some degree of misclassification or over- or underestimation of symptom severity. Future studies incorporating clinician assessments are recommended to enhance data validity. In addition, unmeasured confounding factors, such as prior or post-vaccination medication use, psychological stress, and other health-related variables, may have influenced the results, limiting the accuracy of the conclusions. Larger, clinically validated studies are recommended to strengthen the reliability and applicability of these findings.

## 5. Conclusion

This study examined adverse effects following herpes zoster vaccination and identified different symptom patterns after the first and second doses. After the first dose, fatigue and headache predominated, but chills and injection-site pain increased after the second. Moderate symptoms were the most frequently reported severity category, and the most commonly reported symptom duration was 1–2 days. Medication use to relieve symptoms was more common after the second dose due to a stronger immune response or better preparedness. Zostavax caused more severe reactions than Shingrix, Therefore, balancing the severity of adverse effects with the need for effective immunization is crucial to guide patient counselling and optimize vaccination outcomes in the Saudi population.

There was no gender-based difference in symptom severity. While non-smokers reported slightly more adverse effects than smokers, the difference was not statistically significant. Seniors and those with chronic diseases had more severe symptoms, likely due to immunosenescence and inflammation. These findings emphasize the need for tailored vaccination strategies and pre-vaccination counselling in vulnerable populations to improve safety and manage expectations.

## Supporting information

S1 File This supporting material contains the complete survey instrument used in the current study. (SAV)

S2 File This supplemental material includes the entire study’s survey questionnaire. (XLSX)
