# Supplementary material for: Adverse events of herpes zoster vaccination among adults in Saudi Arabia: A cross-sectional study
Source: PLoS One. 2026 Oct 27;21(10):e0360528. doi: 10.1371/journal.pone.0360528 (PMC13712533; doi:10.1371/journal.pone.0360528)
Supplement: Supplementary file 3 [file pone.0360528.s006.docx]

**Message to the editor**

We sincerely thank you for your careful evaluation of our manuscript and for your constructive comments and valuable suggestions. Your feedback has helped us improve the manuscript’s clarity, methodological presentation, and overall scientific quality. We have carefully addressed all comments and incorporated the corresponding revisions into the manuscript. We hope that the revised version is now suitable for publication.

**Introduction
1. Add more complications, such as meningoencephalitis and cardiovascular complications.**

**Response to the editor:**

Thank you for this valuable suggestion. We have revised the manuscript to include additional complications associated with herpes zoster, particularly meningoencephalitis and cardiovascular complications, such as stroke and myocardial infarction. Relevant references have also been added to support these revisions.

**2. State whether similar publications exist in Saudi Arabia, and describe them along with your improvements.**

**Response to the editor:**

Thank you for this important comment. We have clarified that several related studies have been conducted in Saudi Arabia; however, these publications primarily examined awareness, knowledge, acceptance, uptake, and barriers to herpes zoster vaccination, particularly among older adults and patients with diabetes. To the best of our knowledge, limited Saudi evidence has specifically characterized adverse events following herpes zoster vaccination. Our study extends the existing literature by evaluating vaccinated adults, documenting dose-specific adverse events, their onset, duration, perceived severity, and medication use, and comparing symptom severity across vaccine doses, vaccine types, and demographic and clinical subgroups. These distinctions and the contribution of the present study have now been described in the revised manuscript.

**3. This information is irrelevant because it does not assess HZV; a 2018 U.S. study highlighted serious adverse effects associated with varicella vaccination, including urticaria, ataxia, thrombocytopenia, pneumonia, encephalitis, stroke, and, in rare instances, death.**

**Response to the editor:**

Thank you for this comment. We respectfully consider this information relevant as contextual evidence because both varicella and herpes zoster vaccines target the varicella-zoster virus, and the live-attenuated zoster vaccine contains the same vaccine strain used in the varicella vaccine, although at a higher antigen concentration. Nevertheless, we acknowledge that the cited study evaluated varicella vaccination rather than herpes zoster vaccination and therefore cannot provide direct evidence regarding HZV safety. We have revised the statement to clarify this distinction and avoid attributing these adverse events directly to HZV.

**Methods
1. Study design: This is an overclaim (...in Saudi Arabia) since the study only assessed people in Riyadh, not all of the Saudi population. Please revise this information and the title.**

**Response to the editor:**

Thank you for this comment. We respectfully disagree that the study population was limited to Riyadh. The study was coordinated from Riyadh; however, the online questionnaire was distributed through social media platforms and was open to eligible adults residing throughout Saudi Arabia who had received the her]pes zoster vaccine. Therefore, “Saudi Arabia” refers to the geographical eligibility of the study population and recruitment scope, rather than claiming that the sample statistically represents every vaccinated individual in the country. To prevent misinterpretation, we have clarified the nationwide online recruitment method and acknowledged that convenience sampling limits the national representativeness of the findings. Accordingly, we believe that retaining “Saudi Arabia” in the title is appropriate.

**2. Inclusion: …had been administered the herpes zoster vaccine (are patients with uncompleted schedules included, or is it strictly patients with completed vaccinations)?**

**Response to the editor:**

Thank you for requesting clarification. The study included strictly participants who had completed the recommended herpes zoster vaccination schedule. Individuals with incomplete vaccination schedules were excluded. We have revised the inclusion and exclusion criteria in the Methods section to state this requirement explicitly.

**3. Adverse event data were gathered using a well-organized questionnaire. Validation and reliability testing should be reported. The current validation technique is insufficient.**

**Response to the editor:**

Thank you for this comment. We have clarified the questionnaire development and validation process in the Methods section. The questionnaire was adapted from a previously published study to meet the objectives of the present research [20]. The draft was subsequently reviewed by a principal investigator with expertise in vaccine research, who evaluated its content, wording, and structure. Following refinement based on this feedback, the revised questionnaire was pilot-tested among 20 individuals representative of the target population to assess its clarity, comprehensibility, and relevance. Minor wording modifications were made based on the pilot participants’ feedback before the final questionnaire was distributed.

**4. Power calculation for sample size should be reported alongside 95% CI.**

**Response to the editor**

Thank you for this comment. The sample-size calculation and the corresponding 95% confidence level were already reported in the Methods section. The calculation was performed using the Raosoft sample-size calculator, assuming a 95% confidence level, a 5% margin of error, and a 50% response distribution. This information has been retained and further clarified in the revised manuscript.

**5. Please state sampling methodology (random or non-random). Are there any further inclusion and exclusion criteria?**

**Response to the editor:**

Thank you for this comment. The study included strictly participants who had completed the recommended herpes zoster vaccination schedule. We have expanded and clarified the inclusion and exclusion criteria in the revised manuscript.

**6. The pilot interview includes 20 individuals representative of the target study population. How can the authors ensure about the sample's representativeness? Gender, education, profile, etc? Please describe more.**

**Response to the editor:**

Thanks for this comment. The development and validation procedure of the questionnaire has been explained in the Methods section. The questionnaire was altered from a previously published study to achieve the goals of the current research [20]. A lead investigator with expertise in vaccination research then read the document and assessed the substance, phrasing and structure. The updated questionnaire was piloted on 20 individuals typical of the target demographic to measure clarity, comprehensibility and relevance and then refined on the basis of this feedback. The final questionnaire was given after slight wording changes based on participants’ input in the pilot study.

**7. Please attach the questionnaire given to participants as supplementary material (including the instructions).**

**Response to the editor:**

Thank you for this suggestion. We have attached the complete questionnaire administered to participants, including the introductory information, participant instructions, informed-consent statement, and all questionnaire items and response options, as supplementary material (Supplementary File).

**8. Is there any standard on how the adverse events are assessed? Mild, moderate, and severe definitions should be described thoroughly.**

**Response to the editor**

Thank you. We clarified that adverse-event severity was assessed using a researcher-developed self-reported ordinal item with four response categories: no symptoms, mild, moderate, and severe. No external clinical grading standard or clinician-assigned criteria were used. The categories reflected participants’ overall perceived symptom severity and were not defined by predefined clinical or functional thresholds. This limitation is also acknowledged in the manuscript.

**9. ...compare severity scores across multiple age groups. Please state the source of how patients are clustered based on age group.**

**Response to the editor**

Thank you. We clarified that the original age groups were predefined questionnaire categories. For the additional multivariable analysis, age was dichotomized as ≤50 versus >50 years. Because the original questionnaire included a 41–50-year category, an exact <50 versus ≥50 classification could not be reconstructed without misclassification.

**10. Add post-hoc analysis for Kruskal-Wallis.
response to the edtor**

Thank you. Dunn’s pairwise post-hoc test with Bonferroni adjustment was added following the significant first-dose Kruskal–Wallis test. Significant differences were identified between the 18–25-year group and the 31–40, 41–50, and >60-year groups. No post-hoc analysis was performed for the second dose because the overall Kruskal–Wallis test was not statistically significant.

**Results
1. The authors just repeat information in the table, which is an unfavorable practice. Please summarize the information from... As shown in Table 1, the gender distribution was nearly equal, with 193 females (50.5%) and 189 males (49.5%).**

**Response to the editor:**

Thank you. The Results text preceding Table 1 was substantially shortened to avoid repeating the detailed information already presented in the table. The revised text now summarizes only the main participant and vaccination characteristics, while the complete descriptive information remains in Table 1.

**2. Table 2 should be analyzed statistically since some parameters such as feeling tired (revise to fatigue), headache, temperature or chills (please revise this classification since it is vague), and no adverse events may have differences (based on percentage).**

**Response to the editor**

Thank you. We added paired statistical comparisons restricted to the 152 participants who received both doses, thereby avoiding comparisons between the full first-dose sample and the second-dose subgroup. McNemar’s test was used for paired binary adverse-effect outcomes and medication use, the Stuart–Maxwell test for symptom-onset distributions, and the Wilcoxon signed-rank test for ordinal severity and symptom duration. Holm adjustment was applied to the symptom-specific comparisons. Because some symptoms were collected as combined response categories in the original questionnaire, they could not be separated retrospectively. Their labels were clarified as “Fatigue/headache” and “Fever/chills.”

**3. The data in Tables 2 and 3 do not match regarding adverse events (please recheck since it is confusing and just a repetition). Furthermore, tables 2 and 3 stated relatively similar topics that are complementary to each other. Please merge Tables 2 and 3, and include a statistical analysis as well.
1. Table 2: No adverse effects: 112 (29.3%), 28 (18.4%)
2. Table 3: No symptoms: 115 (30.1%) and 28 (18.4%)
3. Table 2 onset No symptoms 121 (31.7%) 30 (19.7%)
4. Table 3 duration: No symptoms 116 (30.4%) 30 (19.7%).**

**Response to the editor**

Thank you. The original Tables 2 and 3 were merged into a single table presenting adverse effects, symptom onset, self-reported severity, symptom duration, and medication use. We also clarified that “No adverse effects” and the “No symptoms” responses for onset, severity, and duration were obtained from separate questionnaire items, which explains the small differences in their frequencies. Appropriate paired statistical comparisons were added for participants who received both doses.

**4. Add types of statistical analysis below each table.**

**Response to the editor**

Thank you. The statistical tests used for each analysis are now specified in the corresponding table notes, together with the significance threshold.

**5. … using the Mann–Whitney U test and the Kruskal–Wallis test. To provide a more informative interpretation of the results, effect sizes were dividing it into two groups and analyzing it for multivariate regression alongcalculated for the non-parametric comparisons. For Mann–Whitney U tests, effect size r was computed as |Z|/√N, and for the Kruskal–Wallis test, epsilon-squared (ε²) was reported. (Not important in the result; can be added in the methods).**

**Response to the editor**

Thank you. The effect-size calculation procedures were moved to the Statistical Analysis subsection. The Results now report the effect-size estimates without repeating the calculation formulas.

**6. Table 4 -> assess composite data also (regardless of dose or smoking status).**

**Response to the editor**

**7. Do the analysis on the total number of adverse events, regardless of doses (in addition to currently available data).**

**Response to the editor (6&7).**

Thank you. We added an overall descriptive measure of adverse-event burden regardless of dose. The composite burden was operationalized as the total number of specific adverse-effect symptoms reported across the available dose-specific checklists for each participant. Across both doses, 672 individual symptom reports were recorded, comprising 471 after the first dose and 201 after the second dose. Overall, 272 participants (71.2%) reported at least one specific adverse-effect symptom across one or both doses, with a median of 1 reported symptom per participant (IQR: 0–3).

**8. For p-value, under the table, add "*" for significant results, and state the threshold and statistical tests. Do not add statistical tests in the table title.**

**Response to the editor**

Thank you. Table titles were revised to remove the names of statistical tests. The statistical tests used are now specified in the corresponding table notes, and statistically significant results are marked with an asterisk, with *p < 0.05 defined as the significance threshold.

**9. Tables 4 to 7 can be merged and interpreted simultaneously.**

**Response to the editor**

Thank you. The original Tables 4–7 were consolidated into a single table presenting the bivariate comparisons of self-reported severity according to gender, reported vaccine type, smoking status, and chronic disease status. This reduced repetition and allowed the findings to be interpreted together.

**10. A multivariate analysis (logistic regression) is highly recommended to determine which factors are the most significant contributors while highlighting the confounders.**

**Response to the editor**

Thank you. We added a multivariable ordinal logistic regression model because the severity outcome consisted of four ordered categories. The model included gender, age, smoking status, chronic disease status, immune-related disease status, previous herpes zoster diagnosis, and reported vaccine type. Adjusted odds ratios with 95% confidence intervals were reported, and the proportional-odds assumption and multicollinearity were assessed. Previous herpes zoster diagnosis and chronic disease remained independently associated with greater first-dose severity. Importantly, the significant unadjusted association between reported vaccine type and severity was no longer statistically significant after multivariable adjustment, highlighting the importance of accounting for potential confounding.

**11. Where is the post-hoc information on table 8?**

**Response to the editor**

Thank you. Dunn–Bonferroni post-hoc results are now reported following the significant first-dose Kruskal–Wallis test and included in the table note. Significant differences were observed between participants aged 18–25 years and those aged 31–40 years, 41–50 years, and >60 years. No post-hoc testing was performed for the second dose because the overall test was not significant.

**12. For age, consider also dividing it into two groups and analyzing it for multivariate regression also with other parameters (tables 4-7). Search for the most appropriate classification (such as >=50 or <50) based on the available literature.**

**Response to the editor**

Thank you. Age was included in the multivariable model as ≤50 versus >50 years. This was the closest dichotomous classification that could be reconstructed accurately from the original grouped age data. An exact <50 versus ≥50 classification was not possible because participants aged 41–50 years were recorded as a single category.
 **DISCUSSION
1. The second paragraph should be divided into two or three paragraphs given its long form. Also, there is inadequate citation on it, particularly in the first 15+ lines. Please add more citations to any part of the statement or claim.**

**Response to the editor:**

Thank you for this valuable suggestion. We reviewed the statements and claims throughout this section and added relevant supporting citations. These references support the discussion of vaccination patterns, immune-related conditions, vaccine misclassification, vaccine literacy, previous herpes zoster infection, and post-vaccination adverse effects. All corresponding changes have been incorporated into the revised manuscript.

**2. Inconsistent citation style: Similar gaps in vaccine literacy have been documented in prior studies such as Hurley et al. (2018).**

**Response to the editor:**

Thank you for identifying this inconsistency. We have revised the sentence for greater clarity and corrected the citation to conform to the journal’s required referencing style. We have also reviewed the manuscript to ensure consistency in citation formatting throughout.

**3. Following multivariate regression, revise this section. State that from univariate or bivariate perspectives, they can have a significant result. However, in multivariate analyses, certain interactions may be eliminated.**

**Response to the editor:**

Thank you. The Discussion was revised to distinguish the unadjusted bivariate findings from the adjusted multivariable results. In particular, although reported vaccine type was significantly associated with severity in the bivariate analysis, this association was no longer statistically significant after adjustment. The Discussion now emphasizes the independent associations of previous herpes zoster diagnosis and chronic disease with first-dose severity.

**4. Why are most participants aged 18–40 years? The CDC guideline mostly targets this vaccination for people aged >50.**

**Response to the editor:**

Thank you for this important comment. Although the CDC primarily recommends routine herpes zoster vaccination for immunocompetent adults aged ≥50 years, the Saudi Ministry of Health recommends the vaccine in Saudi Arabia for adults aged ≥50 years and adults aged ≥18 years who have an immune-related condition or are receiving immunosuppressive treatment. Therefore, vaccinated individuals aged 18–40 years were eligible for inclusion in our study. Their predominance may also be explained by the online distribution of the questionnaire through social media, which may have resulted in greater participation by younger adults. We have clarified the Saudi eligibility criteria and acknowledged the potential age-related selection bias in the revised manuscript.

**5. Sampling may be flawed. How can the authors arrive at 382 samples in the final analysis? People who filled out the forms are included, as this is very commonly done.
response to the editor:**

Thank you for this comment. In the present study, is utilized in online cross-sectional surveys. The questionnaire was sent electronically to the target participants and those who willingly completed it were assessed against the eligibility criteria set. The final sample consisted of 382 respondents who provided informed consent, indicated received the herpes zoster vaccine, met the inclusion criteria, and provided complete and eligible responses. We have clarified the participant recruiting, response-screening procedure and sample approach in the revised approach section. We also note that convenience sampling can lead to selection bias and restrict the generalizability of the findings.

**CONCLUSION
1. The conclusion is very stretched. Please focus on the main objective and do not describe too much of the study procedure. State several main results, particularly from regression statistics.**

**Response to the editor:**

Thank you for this valuable comment. We reduced and refocused the Conclusion to match the study's main goal. After removing procedural details, the revised conclusion summarizes the main adverse events and highlights the regression analysis's primary conclusions, particularly the significant factors linked with adverse-effect intensity.

**GENERAL
1. Some parts of the manuscript look refined or created through AI assistance. Please declare it as a statement of AI DISCLOSURE AND DECLARATION (please check PLoS One guideline).**

**Response to the editor:**

Thank you for raising this matter. We consulted all co-authors regarding the use of artificial intelligence in preparing the manuscript, and each author confirmed that they did not use AI tools to generate or write its content. All authors have reviewed the manuscript and take full responsibility for its accuracy, integrity, and originality.

**Reviewer #2: I have some suggestions:
1. English proofreading is required.**

**Response to the reviewer:**

Thank you for this comment. The manuscript has undergone comprehensive English-language proofreading to improve its grammar, clarity, readability, and academic style. Typographical errors and inconsistencies have also been corrected throughout the revised manuscript.

**2. The high percentage of participants who were unable to identify the vaccine type may affect the validity of comparisons between Zostavax and Shingrix. (Add this to the limitation section).
Response to the reviewer:**

Thank you for highlighting this issue. This concern has been explicitly addressed in the Limitations section of the revised manuscript. We acknowledge that the high proportion of participants who could not identify their vaccine type may have introduced misclassification and reduced the validity of comparisons between Zostavax and Shingrix. Accordingly, we have stated that the observed differences between the two vaccine groups should be interpreted with caution.
