# Supplementary material for: Adverse events of herpes zoster vaccination among adults in Saudi Arabia: A cross-sectional study
Source: PLoS One. 2026 Oct 27;21(10):e0360528. doi: 10.1371/journal.pone.0360528 (PMC13712533; doi:10.1371/journal.pone.0360528)
Supplement: Supplementary file 4 [file pone.0360528.s003.docx]

Overall, there is a need for English language and grammar check throughout the manuscript.

Title.

The title may be written in the sentence case preferably.

Abstract.

22.3% can not be considered as majority of population. Rewrite the sentence.

Results may be rewritten with clarity for understanding.

No significant gender difference were found… for what?

2.2. Study population

The study include the population residing in Saudi Arabia, however, results also shows the participants as non-saudi (4.5%). Why they were not excluded?

2.5 sample size calculation.

What was the population portion was kept in Raosoft formulaa to determine the sample of 382?

2.6 Study Questionnaire Development.

Abstract clearly indicated a bilingual questionnaire use. However, there is no such information in the methodology section. How the translation was done, which languages were used for questionnaire?

As mentioned in study population, participants residing in Saudi Arabia were included. However, in this section the population was restricted to Riyad, Saudi Arabia? Moreover, how the participants were constraints to Saudi by posting the questionnaire on twitter and other social media plateforms?

It is mentioned that principal investigator provided feedback, wording, ad structure of questionnaire. However, this approach introduces the biasness in the study. It would be better to use a previously available research questionnaire and cite it or it was mandatory to develop the questionnaire and face and content validation could have been done and protocol would be published before the actual study.

Therefore, the study show potential risk of bias.

Statistical analysis.

How the severity scale has been derived? Or it has been adopted from any previous study?

Results.

Describe the results in the uniform pattern, either use N (%) or (N;%) for clarity.

The majority of participants were in the 18–40 age range… mention the percentage of majortiy of participants.

Table 2.2 Which symptoms maybe considered as mild, moderate, severe etc. Whether it based on the participants personal judgement or principal investigators judgment?
