# Supplementary material for: Adverse events of herpes zoster vaccination among adults in Saudi Arabia: A cross-sectional study
Source: PLoS One. 2026 Oct 27;21(10):e0360528. doi: 10.1371/journal.pone.0360528 (PMC13712533; doi:10.1371/journal.pone.0360528)
Supplement: Supplementary file 5 [file pone.0360528.s005.docx]

**Dear Reviewer #1:**

Thank you for taking the time to review my manuscript. I sincerely appreciate your valuable feedback and thoughtful comments. I have completed the revision and addressed all the comments in my manuscript.

1. Study timeline and internal consistency
The reported survey period (6 September 2024 to 15 August 2025) is incompatible with the current submission and review timeline, and therefore, not credible as written. This discrepancy must be corrected and clarified, and all dates in the manuscript must be checked for consistency (including references with future publication years).

**Response to the reviewer**

Thank you for your comment. We apologize for the error in the reported study timeline. The survey period has been corrected, and all dates in the manuscript have been reviewed to ensure internal consistency. In addition, references have been checked and updated to ensure accuracy and appropriate publication years.

**2. Target population and age distribution
Herpes zoster vaccination is generally recommended for older adults and specific high‑risk groups; yet the majority of your sample lies in the 18–40 age range, with 18–25 years being the largest group, and 95% reported as having no immune disease. This pattern is not aligned with usual clinical indications for HZ vaccination.**

**Response to the reviewer**

Thank you for your comment. We apologize for the error in the reported study timeline. The survey period has been corrected, and all dates in the manuscript have been reviewed to ensure internal consistency. In addition, references have been checked and updated to ensure accuracy and appropriate publication years.

**You must clearly explain why such a high proportion of young, apparently healthy adults received HZ vaccination (e.g., immunocompromised status, occupational indication, special institutional policies) and how eligibility was defined and verified. Otherwise, there is a substantial risk that participants misclassified the vaccine (e.g., confusing the HZ vaccine with varicella or another vaccine).**

**Response to the reviewer**

Thank you for this valuable comment. The inclusion of younger participants likely reflects local practices, including prior infection, chronic conditions, or occupational indications. We have clarified in the Methods section that the questionnaire distinguished herpes zoster from other vaccines, and we further emphasized potential misclassification bias in the Discussion. These revisions have been added to the Methods and Discussion sections **3. Verification of vaccination status and vaccine type
Nearly half of the participants were unable to identify which HZ vaccine they had received, yet you perform comparative analyses between Zostavax and Shingrix and draw firm conclusions. This is not methodologically robust.
The high reported prevalence of Zostavax use during the stated period also requires validation, given current practice patterns and the increasing dominance of Shingrix. At minimum, you should describe any steps taken to verify vaccination status and vaccine type (e.g., vaccination cards, electronic records, Sehhaty), and explicitly acknowledge the limitations due to self‑report and “don’t know” responses.**

**Response to the reviewer**

I appreciate your crucial opinion. We agree that the significant proportion of participants who could not identify their HZ vaccine type is a methodological restriction that may weaken comparative analyses between Zostavax and Shingrix.

We clarified vaccination status and vaccine type assessment in the updated text. immunization information was self-reported; immunization cards, electronic medical records, and Sehhaty were not available. We now explicitly address this shortcoming in the text, specifically recollection bias and misclassification due to “don’t know” responses.

Due to current practice patterns and the rising usage of Shingrix, we believe that the reported prevalence of Zostavax use during the study period should be regarded with caution. Thus, we have toned down our conclusions and stressed that any comparison between Zostavax and Shingrix should be exploratory rather than definitive.

The manuscript's Limitations reflect these changes.

4. Findings that contradict established evidence
Your analyses indicate that Zostavax is associated with more severe adverse events than Shingrix. This directly contradicts the well‑documented higher reactogenicity of Shingrix in clinical trials and post‑marketing data. Such a result requires very careful handling: you should
• Compare your findings explicitly with the pivotal trials and existing literature.
• Discuss plausible explanations (e.g., misclassification of vaccine type, selection bias, differential recall).
• Mark these comparisons as exploratory and avoid strong causal or comparative safety claims in the presence of major classification uncertainty.

**Response tpo the reviewer**

Thank you for this important and insightful comment. We agree that the observed finding of higher self-reported severity among participants who reported receiving Zostavax requires careful interpretation, particularly because it does not fully align with the broader evidence base indicating greater reactogenicity with Shingrix in pivotal trials and post-marketing studies. In the revised manuscript, we explicitly compared our finding with the published literature and clarified that this vaccine-type comparison should be considered exploratory rather than confirmatory.

We also expanded the Discussion to address several plausible explanations for this discrepancy. Specifically, we noted the possibility of vaccine-type misclassification, given that a substantial proportion of participants were unable to identify the vaccine they had received. We further acknowledged the potential influence of retrospective self-reporting, including differential recall of both vaccine type and adverse effects, as well as selection bias related to the non-probability sampling approach. In addition, we noted that participant characteristics associated with symptom severity, such as age and chronic disease status, may have differed across vaccine groups.

Finally, we revised the wording in both the Discussion and Conclusion to avoid strong causal or comparative safety claims. The manuscript now presents this finding more cautiously as a self-reported association observed in this sample, rather than as a definitive comparative safety conclusion between the two vaccines.

**5. Methods: sampling, questionnaire, and bias
The sampling strategy (online Google Form distributed via social media) is only briefly described and does not adequately address potential selection, recall, and information biases. You should clearly describe:
The recruitment channels, target population, and approximate response rate.
Inclusion criteria and how you ensured that only genuinely vaccinated individuals responded.
Any steps for questionnaire development, validation, and pilot testing, in a more structured form (content validity, language, and reliability, if available).
Given the design, the risk of recall bias (for both symptoms and vaccine brand) is high and should be explicitly discussed and reflected in the strength of the conclusions.**

**Response to the reviewer**

Thank you for this valuable and insightful comment. We appreciate the reviewer’s observations regarding the need for further clarification of the sampling strategy, questionnaire methodology, and potential biases. In response, we have revised the manuscript accordingly. Specifically:

‏1. We expanded the Methods section to provide a clearer description of the recruitment channels, specifying that the questionnaire was distributed electronically via email, Twitter, Instagram, and WhatsApp, and clarified the target population and eligibility criteria.

‏2. We further clarified the questionnaire development process, including its development based on literature review and expert input, bilingual preparation in English and Arabic, forward and backward translation, principal investigator review for face and content validity, and pilot testing among 20 representative individuals prior to final distribution.

‏3. We strengthened the Limitations section to more explicitly discuss the potential for recall bias related to retrospective self-reported symptoms and vaccine brand recall.

‏These revisions have been incorporated into the revised manuscript to improve methodological clarity and transparency.

6, Results presentation and clarity
Several tables (1.1, 1.2, 1.3, and 2.1, 2.2) contain overlapping content and could be streamlined to reduce redundancy and improve clarity. Percentages that pertain only to subgroups (e.g., n=152 second‑dose recipients) must be clearly labeled in both titles and footnotes to avoid misinterpretation.
Figure 1 is cited but not appropriately presented or described. Ensure that all figures and tables are consistently numbered, fully labeled, and formatted according to journal guidelines.
Where non‑parametric tests are used, consider reporting effect sizes and confidence intervals, not only p‑values, to allow a more meaningful interpretation of differences.

**Response to the reviewer**

Thank you for this valuable suggestion. In response, we revised the presentation of the Results section to reduce redundancy and improve clarity. The original five descriptive tables were consolidated into three tables: one summarizing participant characteristics, health status, and vaccination history; one presenting adverse effects and symptom onset after the first and second doses; and one presenting self-reported severity, symptom duration, and medication use. This revision removed overlapping content across the original tables and provided a clearer structure for the descriptive findings.

We also made subgroup denominators explicit throughout the revised tables. Variables specific to second-dose recipients are now clearly identified, and the corresponding percentages are labeled as being calculated from the subgroup of participants who received a second dose (n = 152). In addition, the previous figure was removed because it repeated information already presented in the revised tables. Table numbering, titles, and notes were also standardized across the manuscript to improve consistency and adherence to journal style.

**Figure 1 is cited but not appropriately presented or described. Ensure that all figures and tables are consistently numbered, fully labeled, and formatted according to journal guidelines.**

**Response to the reviewer**

Thank you for this important comment. To improve clarity and avoid redundancy, Figure 1 was removed from the revised manuscript because it repeated severity data already presented in the descriptive tables. The corresponding findings are now presented only in the revised tables and described directly in the Results section.

We also revised the numbering, titles, and formatting of all tables for consistency across the manuscript. The original descriptive tables were reorganized into three clearly labeled tables, and subgroup-specific results, particularly those related to second-dose recipients, are now explicitly identified in both the table content and notes. These revisions were made to improve readability and better align the presentation with journal formatting expectations.

**Where non‑parametric tests are used, consider reporting effect sizes and confidence intervals, not only p‑values, to allow a more meaningful interpretation of differences.**

**Response to the reviewer**

Thank you for this valuable suggestion. In the revised manuscript, we added effect size estimates to all non-parametric analyses to provide a more informative interpretation of the observed differences beyond p values alone. Specifically, effect size r was reported for all Mann–Whitney U tests, and epsilon-squared (ε²) was reported for the Kruskal–Wallis test. These values were added to the corresponding tables and incorporated into the accompanying text to clarify the magnitude of the observed group differences.

In addition, the Results section was revised so that the interpretation of each comparison now reflects both statistical significance and effect size. This allowed us to distinguish between statistically significant findings with modest effects and those with stronger practical magnitude.

**• Standardize p‑value formatting throughout the manuscript and clarify in the Abstract what specific comparisons significant p‑values refer to.**

**Response to the reviewer**

Thank you for this helpful comment. We standardized p-value formatting throughout the revised manuscript using a consistent style with leading zeros for exact values and p < 0.001 where appropriate. In addition, we revised the Abstract to clarify that the reported significant p values refer specifically to the comparison in self-reported severity between participants who received Zostavax and those who received Shingrix after the first and second doses.

**7. Interpretation, discussion, and limitations
The Discussion section does not sufficiently confront the central limitations of the study: the age distribution that is not representative of the recommended HZ vaccine target group, the high rate of unknown vaccine type, and the reliance on self‑report. These must be discussed clearly and prominently, with a corresponding tempering of the conclusions.
Attempts to justify the young sample by “occupational factors” are not adequately support and should either be substantiated with data or removed.
The limitations section, while present, should be made more concise but also more explicit regarding the impact of sampling and misclassification on external validity and on any comparative conclusions between vaccine brands.
Conclusions and public health implications must be reframed to reflect that the findings primarily describe a young adult, self‑selected, and potentially misclassified sample, rather than the typical older adult population for whom HZ vaccination is indicated.**

**Response to the reviewer**

Thank you for this important comment. The reference to occupational factors has been removed as it was not supported by our data. The Limitations section has been revised to be more concise and to clearly address the impact of sampling and potential misclassification on external validity and vaccine comparisons. In addition, the Conclusion has been updated to reflect that the findings are based on a younger, self-selected, and self-reported sample.

8. Technical and editorial issues
• Ensure all dates (study period, reference years) are realistic and internally consistent.
• Standardize p‑value formatting throughout the manuscript and clarify in the Abstract what specific comparisons significant p‑values refer to.
• Check all references for completeness, consistency with journal style, and plausibility of publication dates.

**Response to the reviewer**

Thank you for your comment. We have corrected the study timeline and reviewed all dates for consistency. P-values have been standardized, and the Abstract has been clarified. References were also checked and updated as needed.

**Reviewer #2:**

I am appreciative of the time you set down to read through my manuscript. Please know that I am extremely grateful for your insightful remarks and important feedback. All of the criticisms that were made in my document have been handled, and I have finished the rewrite.

**The abstract is clear but requires minor grammatical editing.**
response to the reviewer

Thank you for your comment. We appreciate your positive assessment of the abstract. The abstract has been carefully revised and edited for grammar, clarity, and readability.

The questionnaire validation process needs more methodological detail (2-3 sentences).

**Response to the reviewer**

Thank you for this helpful comment. Additional details have been added to the Methods section to further clarify the questionnaire validation process.

**The Data Availability statement (“available upon request”) may not meet PLOS policy (check upon re submission)
response to the reviewer**

Thank you for highlighting this point. We updated the statement and thoroughly review the journal's data sharing policy upon resubmission to assure PLOS compliance.

**Statistical methods are appropriate and clearly justified.**

**response to the reviewer**

Thank you for your positive comment. We appreciate your assessment that the statistical methods were appropriate and clearly justified.

**Add more specific policy recommendations in the conclusion section.**

**response to the reviewer**

Thank you for this valuable suggestion, we have revised the conclusion section to include more specific. These now emphasise strengthening public awareness initiatives, improving physician-led vaccine counseling, expanding access to herpes zoster vaccination, and supporting national strategies that promote adult immunization uptake among high-risk populations

**Reviewer #3:**

I appreciate your consideration of my manuscript. I genuinely value your insightful criticism and considerate remarks. I have finalized the revision and responded to all suggestions in my text.

**First, the manuscript requires comprehensive English language editing. Numerous grammatical and structural errors reduce clarity and hinder interpretation of the findings. Careful proofreading or professional language editing is strongly recommended.**

**Regarding the title, consistency with journal style should be considered, and sentence case formatting is recommended.**

**Response to the reviewer**

Thank you for your comment. The title has been revised to follow sentence case formatting and to align with the journal’s style guidelines.

**In the abstract, several statements require correction or clarification. The claim that 22.3% represents a majority of the population is inaccurate and should be revised. The Results section would benefit from clearer presentation of key outcomes. Additionally, the statement indicating no significant gender difference is incomplete and should specify the outcome variable being compared.

The description of the study population requires clarification. The methodology states that participants residing in Saudi Arabia were included; however, the results indicate that a proportion of participants were non-Saudi. The authors should clarify eligibility criteria and explain whether nationality or residency was the inclusion criterion. If residency was the basis for inclusion, this should be clearly stated. Furthermore, inconsistencies appear between the stated national scope of recruitment and references to recruitment from Riyadh. This discrepancy should be resolved.**

**Response to the reviewer**

Thank you for this valuable comment. We appreciate the reviewer’s observation regarding the need to clarify the study population. We would like to clarify that the inclusion criterion was residency in Saudi Arabia rather than Saudi nationality. Therefore, both Saudi and non-Saudi individuals residing in Saudi Arabia were eligible for participation. The manuscript has been revised accordingly to clearly state this eligibility criterion and to ensure consistency regarding the national scope of recruitment throughout the manuscript. **The sample size calculation requires greater transparency. The parameters used in the Raosoft calculation, including assumed population proportion, confidence level, and margin of error, should be explicitly reported to allow reproducibility.

The questionnaire development process requires more detailed methodological description. The abstract indicates use of a bilingual questionnaire; however, translation procedures, languages used, and validation steps are not described in the methods section. The authors should clarify how translation was conducted and whether forward–backward translation or pilot testing was performed. Additionally, reliance on investigator input alone in questionnaire design may introduce measurement bias. Information on face validity, content validity, pilot testing, or reliability assessment should be provided. If the instrument was adapted from previous research, appropriate citation should be included. The use of online recruitment via social media platforms raises potential concerns regarding selection bias and geographic eligibility control; these issues should be acknowledged and discussed.**

**Response to the reviewer**

Thank you for this valuable comment. We have revised the questionnaire development section to provide additional methodological details. Specifically, we clarified that the questionnaire was initially developed in English and translated into Arabic using forward and backward translation to ensure linguistic consistency. Furthermore, the questionnaire underwent pilot testing among 20 representative individuals to assess clarity, comprehension, and relevance before final distribution. **The statistical analysis section requires clarification regarding the derivation of the adverse event severity scale. The authors should specify whether severity categories were self-reported or based on predefined criteria. If the scale was adopted from prior literature, a reference should be provided; if developed for this study, justification and operational definitions are necessary.**

**Response to the reviewer**

Thank you for this important comment. We have clarified the derivation of the adverse event severity measure in the revised manuscript. The severity variable was not adopted from a previously validated external scale. Rather, it was obtained from the researcher-developed questionnaire as a direct self-reported ordinal item for each vaccine dose. Participants rated the overall severity of their post-vaccination side effects using four predefined response categories: no symptoms, mild, moderate, and severe. Thus, the severity categories were self-reported by participants and were not assigned by clinicians. We also clarified in the Methods section that this variable reflected participant-perceived symptom severity and was analysed as an ordinal outcome.

**In the results section, presentation should follow a consistent reporting format, such as N (%), throughout the manuscript. Statements referring to a “majority” should include corresponding percentages. Tables require clearer labeling and standardized formatting. Specifically, the classification of symptoms into mild, moderate, and severe requires explicit operational definitions and explanation of how these categories were assigned.**

**Response to the reviewer**

Thank you for this helpful comment. We revised the Results section to ensure a more consistent reporting format throughout the manuscript. Specifically, descriptive findings are now presented more systematically using n (%) in both the text and tables, and statements such as “majority” or “most” were revised to include the corresponding frequencies and percentages. We also reorganized the descriptive presentation of the results by consolidating the original descriptive tables into three clearly labeled tables, which improved clarity and reduced overlap. In addition, subgroup-specific denominators are now explicitly identified, particularly for second-dose recipients (n = 152), in both the tables and their notes. Finally, the classification of symptom severity was clarified in the Methods section and table notes by explicitly stating that severity was measured as a self-reported ordinal item in the researcher-developed questionnaire using predefined categories of no symptoms, mild, moderate, and severe.
