# Supplementary material for: Adverse events of herpes zoster vaccination among adults in Saudi Arabia: A cross-sectional study
Source: PLoS One. 2026 Oct 27;21(10):e0360528. doi: 10.1371/journal.pone.0360528 (PMC13712533; doi:10.1371/journal.pone.0360528)
Supplement: Peer review history

# Peer review: Decision Letter 0

**DOI:** 10.1371/journal.pone.0360528.r001

**Authors:** Godwin Anywar

31 Mar 2026

plosone@plos.org. When you're ready to submit your revision, log on to https://www.editorialmanager.com/pone/ and select the 'Submissions Needing Revision' folder to locate your manuscript file.

- A letter that responds to each point raised by the academic editor and reviewer(s). You should upload this letter as a separate file labeled 'Response to Reviewers'.
- A marked-up copy of your manuscript that highlights changes made to the original version. You should upload this as a separate file labeled 'Revised Manuscript with Track Changes'.
- An unmarked version of your revised paper without tracked changes. You should upload this as a separate file labeled 'Manuscript'.

If you would like to make changes to your financial disclosure, please include your updated statement in your cover letter. Guidelines for resubmitting your figure files are available below the reviewer comments at the end of this letter.

If applicable, we recommend that you deposit your laboratory protocols in protocols.io to enhance the reproducibility of your results. Protocols.io assigns your protocol its own identifier (DOI) so that it can be cited independently in the future. For instructions see: https://journals.plos.org/plosone/s/submission-guidelines#loc-laboratory-protocols. Additionally, PLOS ONE offers an option for publishing peer-reviewed Lab Protocol articles, which describe protocols hosted on protocols.io. Read more information on sharing protocols at https://plos.org/protocols?utm_medium=editorial-email&utm_source=authorletters&utm_campaign=protocols.

As the corresponding author, your ORCID iD is verified in the submission system and will appear in the published article. PLOS supports the use of ORCID, and we encourage all coauthors to register for an ORCID iD and use it as well. Please encourage your coauthors to verify their ORCID iD within the submission system before final acceptance, as unverified ORCID iDs will not appear in the published article. *Only*  the individual author can complete the verification step; PLOS staff *cannot*  verify ORCID iDs on behalf of authors.

We look forward to receiving your revised manuscript.

Kind regards,

Godwin Upoki Anywar, BSc, Msc, PhD

Academic Editor

PLOS One

Journal Requirements:

When submitting your revision, we need you to address these additional requirements.

1. Please ensure that your manuscript meets PLOS ONE's style requirements, including those for file naming. The PLOS ONE style templates can be found at

https://journals.plos.org/plosone/s/file?id=wjVg/PLOSOne_formatting_sample_main_body.pdf and

https://journals.plos.org/plosone/s/file?id=ba62/PLOSOne_formatting_sample_title_authors_affiliations.pdf

2. Please provide additional details regarding participant consent. In the ethics statement in the Methods and online submission information, please ensure that you have specified what type you obtained (for instance, written or verbal, and if verbal, how it was documented and witnessed). If your study included minors, state whether you obtained consent from parents or guardians. If the need for consent was waived by the ethics committee, please include this information.

Once you have amended this/these statement(s) in the Methods section of the manuscript, please add the same text to the “Ethics Statement” field of the submission form (via “Edit Submission”).

For additional information about PLOS ONE ethical requirements for human subjects research, please refer to http://journals.plos.org/plosone/s/submission-guidelines#loc-human-subjects-research.

3. Please update your submission to use the PLOS LaTeX template. The template and more information on our requirements for LaTeX submissions can be found at http://journals.plos.org/plosone/s/latex.

4. Thank you for stating the following in the Acknowledgments Section of your manuscript:

“This work was supported and funded by the Deanship of Scientific Research at Imam Mohammad Ibn Saud Islamic University (IMSIU) (grant number IMSIU-DDRSP2501).”

We note that you have provided funding information that is not currently declared in your Funding Statement. However, funding information should not appear in the Acknowledgments section or other areas of your manuscript. We will only publish funding information present in the Funding Statement section of the online submission form.

Please remove any funding-related text from the manuscript and let us know how you would like to update your Funding Statement. Currently, your Funding Statement reads as follows:

“This work was supported and funded by the Deanship of Scientific Research at Imam Mohammad Ibn Saud Islamic University (IMSIU) (grant number IMSIU-DDRSP2501).”

Please include your amended statements within your cover letter; we will change the online submission form on your behalf.

5. Thank you for stating the following financial disclosure:

“This work was supported and funded by the Deanship of Scientific Research at Imam Mohammad Ibn Saud Islamic University (IMSIU) (grant number IMSIU-DDRSP2501).”

Please state what role the funders took in the study.  If the funders had no role, please state: "The funders had no role in study design, data collection and analysis, decision to publish, or preparation of the manuscript."

If this statement is not correct you must amend it as needed.

Please include this amended Role of Funder statement in your cover letter; we will change the online submission form on your behalf.

6. Thank you for stating the following financial disclosure:

“This work was supported and funded by the Deanship of Scientific Research at Imam Mohammad Ibn Saud Islamic University (IMSIU) (grant number IMSIU-DDRSP2501).”

We note that one or more of the authors is affiliated with the funding organization, indicating the funder may have had some role in the design, data collection, analysis or preparation of your manuscript for publication; in other words, the funder played an indirect role through the participation of the co-authors. If the funding organization did not play a role in the study design, data collection and analysis, decision to publish, or preparation of the manuscript and only provided financial support in the form of authors' salaries and/or research materials, please do the following:

1. Review your statements relating to the author contributions, and ensure you have specifically and accurately indicated the role(s) that these authors had in your study. These amendments should be made in the online form.

2. Confirm in your cover letter that you agree with the following statement, and we will change the online submission form on your behalf:

“The funder provided support in the form of salaries for authors [insert relevant initials], but did not have any additional role in the study design, data collection and analysis, decision to publish, or preparation of the manuscript. The specific roles of these authors are articulated in the ‘author contributions’ section.

7. Please provide a complete Data Availability Statement in the submission form, ensuring you include all necessary access information or a reason for why you are unable to make your data freely accessible. If your research concerns only data provided within your submission, please write "All data are in the manuscript and/or supporting information files" as your Data Availability Statement.

8. Please amend either the title on the online submission form (via Edit Submission) or the title in the manuscript so that they are identical.

9. Please amend your authorship list in your manuscript file to include author JEHAD A ALDALI, Emadeldin Elsokkary, Ghadah Alnajdi, Marwa Maghrabi, Ruba Subh, Ghala Alakkasi, Sara Alhanshi Yati and Meshal Alzakari.

10. Your ethics statement should only appear in the Methods section of your manuscript. If your ethics statement is written in any section besides the Methods, please delete it from any other section.

11. If the reviewer comments include a recommendation to cite specific previously published works, please review and evaluate these publications to determine whether they are relevant and should be cited. There is no requirement to cite these works unless the editor has indicated otherwise.

[Note: HTML markup is below. Please do not edit.]

Reviewers' comments:

Reviewer's Responses to Questions

**Comments to the Author**

1. Is the manuscript technically sound, and do the data support the conclusions?

Reviewer #1: Partly

Reviewer #2: Partly

Reviewer #3: Yes

**********

2. Has the statistical analysis been performed appropriately and rigorously?

Reviewer #1: Yes

Reviewer #2: I Don't Know

Reviewer #3: Yes

**********

3. Have the authors made all data underlying the findings in their manuscript fully available?

The PLOS Data policy

Reviewer #1: Yes

Reviewer #2: Yes

Reviewer #3: Yes

**********

4. Is the manuscript presented in an intelligible fashion and written in standard English?

Reviewer #1: Yes

Reviewer #2: Yes

Reviewer #3: No

**********

Reviewer #1: Dear authors

Thank you for submitting your manuscript entitled “Adverse events of herpes zoster vaccination Among Adults in Saudi Arabia: A Cross-Sectional Study.” The study addresses an important and underexplored area in vaccine safety monitoring and has the potential to contribute to understanding adverse events following herpes zoster vaccination in Saudi Arabia. Nevertheless, there are serious conceptual, methodological, and reporting issues that need to be addressed. Below are the major points that require your attention:

1. Study timeline and internal consistency

The reported survey period (6 September 2024 to 15 August 2025) is incompatible with the current submission and review timeline, and therefore, not credible as written. This discrepancy must be corrected and clarified, and all dates in the manuscript must be checked for consistency (including references with future publication years).

2. Target population and age distribution

Herpes zoster vaccination is generally recommended for older adults and specific high‑risk groups; yet the majority of your sample lies in the 18–40 age range, with 18–25 years being the largest group, and 95% reported as having no immune disease. This pattern is not aligned with usual clinical indications for HZ vaccination.

You must clearly explain why such a high proportion of young, apparently healthy adults received HZ vaccination (e.g., immunocompromised status, occupational indication, special institutional policies) and how eligibility was defined and verified. Otherwise, there is a substantial risk that participants misclassified the vaccine (e.g., confusing the HZ vaccine with varicella or another vaccine).

3. Verification of vaccination status and vaccine type

Nearly half of the participants were unable to identify which HZ vaccine they had received, yet you perform comparative analyses between Zostavax and Shingrix and draw firm conclusions. This is not methodologically robust.

The high reported prevalence of Zostavax use during the stated period also requires validation, given current practice patterns and the increasing dominance of Shingrix. At minimum, you should describe any steps taken to verify vaccination status and vaccine type (e.g., vaccination cards, electronic records, Sehhaty), and explicitly acknowledge the limitations due to self‑report and “don’t know” responses.

4. Findings that contradict established evidence

Your analyses indicate that Zostavax is associated with more severe adverse events than Shingrix. This directly contradicts the well‑documented higher reactogenicity of Shingrix in clinical trials and post‑marketing data. Such a result requires very careful handling: you should

• Compare your findings explicitly with the pivotal trials and existing literature.

• Discuss plausible explanations (e.g., misclassification of vaccine type, selection bias, differential recall).

• Mark these comparisons as exploratory and avoid strong causal or comparative safety claims in the presence of major classification uncertainty.

5. Methods: sampling, questionnaire, and bias

The sampling strategy (online Google Form distributed via social media) is only briefly described and does not adequately address potential selection, recall, and information biases. You should clearly describe:

The recruitment channels, target population, and approximate response rate.

Inclusion criteria and how you ensured that only genuinely vaccinated individuals responded.

Any steps for questionnaire development, validation, and pilot testing, in a more structured form (content validity, language, and reliability, if available).

Given the design, the risk of recall bias (for both symptoms and vaccine brand) is high and should be explicitly discussed and reflected in the strength of the conclusions.

6. Results presentation and clarity

Several tables (1.1, 1.2, 1.3, and 2.1, 2.2) contain overlapping content and could be streamlined to reduce redundancy and improve clarity. Percentages that pertain only to subgroups (e.g., n=152 second‑dose recipients) must be clearly labeled in both titles and footnotes to avoid misinterpretation.

Figure 1 is cited but not appropriately presented or described. Ensure that all figures and tables are consistently numbered, fully labeled, and formatted according to journal guidelines.

Where non‑parametric tests are used, consider reporting effect sizes and confidence intervals, not only p‑values, to allow a more meaningful interpretation of differences.

7. Interpretation, discussion, and limitations

The Discussion section does not sufficiently confront the central limitations of the study: the age distribution that is not representative of the recommended HZ vaccine target group, the high rate of unknown vaccine type, and the reliance on self‑report. These must be discussed clearly and prominently, with a corresponding tempering of the conclusions.

Attempts to justify the young sample by “occupational factors” are not adequately support and should either be substantiated with data or removed.

The limitations section, while present, should be made more concise but also more explicit regarding the impact of sampling and misclassification on external validity and on any comparative conclusions between vaccine brands.

Conclusions and public health implications must be reframed to reflect that the findings primarily describe a young adult, self‑selected, and potentially misclassified sample, rather than the typical older adult population for whom HZ vaccination is indicated.

8. Technical and editorial issues

• Ensure all dates (study period, reference years) are realistic and internally consistent.

• Standardize p‑value formatting throughout the manuscript and clarify in the Abstract what specific comparisons significant p‑values refer to.

• Check all references for completeness, consistency with journal style, and plausibility of publication dates.

Overall assessment

The topic is important, and the attempt to document adverse events in the Saudi context is commendable. However, unresolved issues regarding the study population, vaccine verification, and contradictions with established evidence make the current version unsuitable for publication. Addressing the points above would require a deep revision of the study framing, methods description, analysis, and interpretation, and may ultimately necessitate reframing the work as an exploratory, hypothesis‑generating survey with limited generalizability rather than a definitive comparative safety assessment

Sincerely,

Reviewer #2: The abstract is clear but requires minor grammatical editing.

The questionnaire validation process needs more methodological detail (2-3 sentences).

The Data Availability statement (“available upon request”) may not meet PLOS policy (check upon re submission)

Statistical methods are appropriate and clearly justified.

Add more specific policy recommendations in the conclusion section.

Reviewer #3: This manuscript addresses an important public health topic by evaluating adverse events following herpes zoster vaccination among adults residing in Saudi Arabia. The subject is relevant to vaccine safety monitoring and population health. The cross-sectional approach provides useful descriptive insight; however, several methodological, reporting, and clarity issues require substantial revision to ensure transparency, reproducibility, and scientific rigor.

First, the manuscript requires comprehensive English language editing. Numerous grammatical and structural errors reduce clarity and hinder interpretation of the findings. Careful proofreading or professional language editing is strongly recommended.

Regarding the title, consistency with journal style should be considered, and sentence case formatting is recommended.

In the abstract, several statements require correction or clarification. The claim that 22.3% represents a majority of the population is inaccurate and should be revised. The Results section would benefit from clearer presentation of key outcomes. Additionally, the statement indicating no significant gender difference is incomplete and should specify the outcome variable being compared.

The description of the study population requires clarification. The methodology states that participants residing in Saudi Arabia were included; however, the results indicate that a proportion of participants were non-Saudi. The authors should clarify eligibility criteria and explain whether nationality or residency was the inclusion criterion. If residency was the basis for inclusion, this should be clearly stated. Furthermore, inconsistencies appear between the stated national scope of recruitment and references to recruitment from Riyadh. This discrepancy should be resolved.

The sample size calculation requires greater transparency. The parameters used in the Raosoft calculation, including assumed population proportion, confidence level, and margin of error, should be explicitly reported to allow reproducibility.

The questionnaire development process requires more detailed methodological description. The abstract indicates use of a bilingual questionnaire; however, translation procedures, languages used, and validation steps are not described in the methods section. The authors should clarify how translation was conducted and whether forward–backward translation or pilot testing was performed. Additionally, reliance on investigator input alone in questionnaire design may introduce measurement bias. Information on face validity, content validity, pilot testing, or reliability assessment should be provided. If the instrument was adapted from previous research, appropriate citation should be included. The use of online recruitment via social media platforms raises potential concerns regarding selection bias and geographic eligibility control; these issues should be acknowledged and discussed.

The statistical analysis section requires clarification regarding the derivation of the adverse event severity scale. The authors should specify whether severity categories were self-reported or based on predefined criteria. If the scale was adopted from prior literature, a reference should be provided; if developed for this study, justification and operational definitions are necessary.

In the results section, presentation should follow a consistent reporting format, such as N (%), throughout the manuscript. Statements referring to a “majority” should include corresponding percentages. Tables require clearer labeling and standardized formatting. Specifically, the classification of symptoms into mild, moderate, and severe requires explicit operational definitions and explanation of how these categories were assigned.

**********

what does this mean?). If published, this will include your full peer review and any attached files.

If you choose “no”, your identity will remain anonymous but your review may still be made public.

**Do you want your identity to be public for this peer review?**  For information about this choice, including consent withdrawal, please see our Privacy Policy

Reviewer #1: **Yes:**  Mohaned A E;hefny

Reviewer #2: **Yes:**  Nasser M Alorfi

Reviewer #3: No

**********

[NOTE: If reviewer comments were submitted as an attachment file, they will be attached to this email and accessible via the submission site. Please log into your account, locate the manuscript record, and check for the action link "View Attachments". If this link does not appear, there are no attachment files.]

To ensure your figures meet our technical requirements, please review our figure guidelines: https://journals.plos.org/plosone/s/figures

You may also use PLOS’s free figure tool, NAAS, to help you prepare publication quality figures: https://journals.plos.org/plosone/s/figures#loc-tools-for-figure-preparation.

NAAS will assess whether your figures meet our technical requirements by comparing each figure against our figure specifications.

Attachment Submitted filename: Herpez Zoster.docx

# Author comment: Author response to Decision Letter 1

**DOI:** 10.1371/journal.pone.0360528.r002

28 Apr 2026

Dear Reviewer #1:

Thank you for taking the time to review my manuscript. I sincerely appreciate your valuable feedback and thoughtful comments. I have completed the revision and addressed all the comments in my manuscript.

1. Study timeline and internal consistency

The reported survey period (6 September 2024 to 15 August 2025) is incompatible with the current submission and review timeline, and therefore, not credible as written. This discrepancy must be corrected and clarified, and all dates in the manuscript must be checked for consistency (including references with future publication years).

Response to the reviewer

Thank you for your comment. We apologize for the error in the reported study timeline. The survey period has been corrected, and all dates in the manuscript have been reviewed to ensure internal consistency. In addition, references have been checked and updated to ensure accuracy and appropriate publication years.

2. Target population and age distribution

Herpes zoster vaccination is generally recommended for older adults and specific high risk groups; yet the majority of your sample lies in the 18–40 age range, with 18–25 years being the largest group, and 95% reported as having no immune disease. This pattern is not aligned with usual clinical indications for HZ vaccination.

Response to the reviewer

Thank you for your comment. We apologize for the error in the reported study timeline. The survey period has been corrected, and all dates in the manuscript have been reviewed to ensure internal consistency. In addition, references have been checked and updated to ensure accuracy and appropriate publication years.

You must clearly explain why such a high proportion of young, apparently healthy adults received HZ vaccination (e.g., immunocompromised status, occupational indication, special institutional policies) and how eligibility was defined and verified. Otherwise, there is a substantial risk that participants misclassified the vaccine (e.g., confusing the HZ vaccine with varicella or another vaccine).

Response to the reviewer

Thank you for this valuable comment. The inclusion of younger participants likely reflects local practices, including prior infection, chronic conditions, or occupational indications. We have clarified in the Methods section that the questionnaire distinguished herpes zoster from other vaccines, and we further emphasized potential misclassification bias in the Discussion. These revisions have been added to the Methods and Discussion sections

3. Verification of vaccination status and vaccine type

Nearly half of the participants were unable to identify which HZ vaccine they had received, yet you perform comparative analyses between Zostavax and Shingrix and draw firm conclusions. This is not methodologically robust.

The high reported prevalence of Zostavax use during the stated period also requires validation, given current practice patterns and the increasing dominance of Shingrix. At minimum, you should describe any steps taken to verify vaccination status and vaccine type (e.g., vaccination cards, electronic records, Sehhaty), and explicitly acknowledge the limitations due to self report and “don’t know” responses.

Response to the reviewer

I appreciate your crucial opinion. We agree that the significant proportion of participants who could not identify their HZ vaccine type is a methodological restriction that may weaken comparative analyses between Zostavax and Shingrix.

We clarified vaccination status and vaccine type assessment in the updated text. immunization information was self-reported; immunization cards, electronic medical records, and Sehhaty were not available. We now explicitly address this shortcoming in the text, specifically recollection bias and misclassification due to “don’t know” responses.

Due to current practice patterns and the rising usage of Shingrix, we believe that the reported prevalence of Zostavax use during the study period should be regarded with caution. Thus, we have toned down our conclusions and stressed that any comparison between Zostavax and Shingrix should be exploratory rather than definitive.

The manuscript's Limitations reflect these changes.

4. Findings that contradict established evidence

Your analyses indicate that Zostavax is associated with more severe adverse events than Shingrix. This directly contradicts the well documented higher reactogenicity of Shingrix in clinical trials and post marketing data. Such a result requires very careful handling: you should

• Compare your findings explicitly with the pivotal trials and existing literature.

• Discuss plausible explanations (e.g., misclassification of vaccine type, selection bias, differential recall).

• Mark these comparisons as exploratory and avoid strong causal or comparative safety claims in the presence of major classification uncertainty.

Response tpo the reviewer

Thank you for this important and insightful comment. We agree that the observed finding of higher self-reported severity among participants who reported receiving Zostavax requires careful interpretation, particularly because it does not fully align with the broader evidence base indicating greater reactogenicity with Shingrix in pivotal trials and post-marketing studies. In the revised manuscript, we explicitly compared our finding with the published literature and clarified that this vaccine-type comparison should be considered exploratory rather than confirmatory.

We also expanded the Discussion to address several plausible explanations for this discrepancy. Specifically, we noted the possibility of vaccine-type misclassification, given that a substantial proportion of participants were unable to identify the vaccine they had received. We further acknowledged the potential influence of retrospective self-reporting, including differential recall of both vaccine type and adverse effects, as well as selection bias related to the non-probability sampling approach. In addition, we noted that participant characteristics associated with symptom severity, such as age and chronic disease status, may have differed across vaccine groups.

Finally, we revised the wording in both the Discussion and Conclusion to avoid strong causal or comparative safety claims. The manuscript now presents this finding more cautiously as a self-reported association observed in this sample, rather than as a definitive comparative safety conclusion between the two vaccines.

5. Methods: sampling, questionnaire, and bias

The sampling strategy (online Google Form distributed via social media) is only briefly described and does not adequately address potential selection, recall, and information biases. You should clearly describe:

The recruitment channels, target population, and approximate response rate.

Inclusion criteria and how you ensured that only genuinely vaccinated individuals responded.

Any steps for questionnaire development, validation, and pilot testing, in a more structured form (content validity, language, and reliability, if available).

Given the design, the risk of recall bias (for both symptoms and vaccine brand) is high and should be explicitly discussed and reflected in the strength of the conclusions.

Response to the reviewer

Thank you for this valuable and insightful comment. We appreciate the reviewer’s observations regarding the need for further clarification of the sampling strategy, questionnaire methodology, and potential biases. In response, we have revised the manuscript accordingly. Specifically:

‏1. We expanded the Methods section to provide a clearer description of the recruitment channels, specifying that the questionnaire was distributed electronically via email, Twitter, Instagram, and WhatsApp, and clarified the target population and eligibility criteria.

‏2. We further clarified the questionnaire development process, including its development based on literature review and expert input, bilingual preparation in English and Arabic, forward and backward translation, principal investigator review for face and content validity, and pilot testing among 20 representative individuals prior to final distribution.

‏3. We strengthened the Limitations section to more explicitly discuss the potential for recall bias related to retrospective self-reported symptoms and vaccine brand recall.

‏These revisions have been incorporated into the revised manuscript to improve methodological clarity and transparency.

6, Results presentation and clarity

Several tables (1.1, 1.2, 1.3, and 2.1, 2.2) contain overlapping content and could be streamlined to reduce redundancy and improve clarity. Percentages that pertain only to subgroups (e.g., n=152 second dose recipients) must be clearly labeled in both titles and footnotes to avoid misinterpretation.

Figure 1 is cited but not appropriately presented or described. Ensure that all figures and tables are consistently numbered, fully labeled, and formatted according to journal guidelines.

Where non parametric tests are used, consider reporting effect sizes and confidence intervals, not only p values, to allow a more meaningful interpretation of differences.

Response to the reviewer

Thank you for this valuable suggestion. In response, we revised the presentation of the Results section to reduce redundancy and improve clarity. The original five descriptive tables were consolidated into three tables: one summarizing participant characteristics, health status, and vaccination history; one presenting adverse effects and symptom onset after the first and second doses; and one presenting self-reported severity, symptom duration, and medication use. This revision removed overlapping content across the original tables and provided a clearer structure for the descriptive findings.

We also made subgroup denominators explicit throughout the revised tables. Variables specific to second-dose recipients are now clearly identified, and the corresponding percentages are labeled as being calculated from the subgroup of participants who received a second dose (n = 152). In addition, the previous figure was removed because it repeated information already presented in the revised tables. Table numbering, titles, and notes were also standardized across the manuscript to improve consistency and adherence to journal style.

Figure 1 is cited but not appropriately presented or described. Ensure that all figures and tables are consistently numbered, fully labeled, and formatted according to journal guidelines.

Response to the reviewer

Thank you for this important comment. To improve clarity and avoid redundancy, Figure 1 was removed from the revised manuscript because it repeated severity data already presented in the descriptive tables. The corresponding findings are now presented only in the revised tables and described directly in the Results section.

We also revised the numbering, titles, and formatting of all tables for consistency across the manuscript. The original descriptive tables were reorganized into three clearly labeled tables, and subgroup-specific results, particularly those related to second-dose recipients, are now explicitly identified in both the table content and notes. These revisions were made to improve readability and better align the presentation with journal formatting expectations.

Where non parametric tests are used, consider reporting effect sizes and confidence intervals, not only p values, to allow a more meaningful interpretation of differences.

Response to the reviewer

Thank you for this valuable suggestion. In the revised manuscript, we added effect size estimates to all non-parametric analyses to provide a more informative interpretation of the observed differences beyond p values alone. Specifically, effect size r was reported for all Mann–Whitney U tests, and epsilon-squared (ε²) was reported for the Kruskal–Wallis test. These values were added to the corresponding tables and incorporated into the accompanying text to clarify the magnitude of the observed group differences.

In addition, the Results section was revised so that the interpretation of each comparison now reflects both statistical significance and effect size. This allowed us to distinguish between statistically significant findings with modest effects and those with stronger practical magnitude.

• Standardize p value formatting throughout the manuscript and clarify in the Abstract what specific comparisons significant p values refer to.

Response to the reviewer

Thank you for this helpful comment. We standardized p-value formatting throughout the revised manuscript using a consistent style with leading zeros for exact values and p < 0.001 where appropriate. In addition, we revised the Abstract to clarify that the reported significant p values refer specifically to the comparison in self-reported severity between participants who received Zostavax and those who received Shingrix after the first and second doses.

7. Interpretation, discussion, and limitations

The Discussion section does not sufficiently confront the central limitations of the study: the age distribution that is not representative of the recommended HZ vaccine target group, the high rate of unknown vaccine type, and the reliance on self report. These must be discussed clearly and prominently, with a corresponding tempering of the conclusions.

Attempts to justify the young sample by “occupational factors” are not adequately support and should either be substantiated with data or removed.

The limitations section, while present, should be made more concise but also more explicit regarding the impact of sampling and misclassification on external validity and on any comparative conclusions between vaccine brands.

Conclusions and public health implications must be reframed to reflect that the findings primarily describe a young adult, self selected, and potentially misclassified sample, rather than the typical older adult population for whom HZ vaccination is indicated.

Response to the reviewer

Thank you for this important comment. The reference to occupational factors has been removed as it was not supported by our data. The Limitations section has been revised to be more concise and to clearly address the impact of sampling and potential misclassification on external validity and vaccine comparisons. In addition, the Conclusion has been updated to reflect that the findings are based on a younger, self-selected, and self-reported sample.

8. Technical and editorial issues

• Ensure all dates (study period, reference years) are realistic and internally consistent.

• Standardize p value formatting throughout the manuscript and clarify in the Abstract what specific comparisons significant p values refer to.

• Check all references for completeness, consistency with journal style, and plausibility of publication dates.

Response to the reviewer

Thank you for your comment. We have corrected the study timeline and reviewed all dates for consistency. P-values have been standardized, and the Abstract has been clarified. References were also checked and updated as needed.

Reviewer #2:

I am appreciative of the time you set down to read through my manuscript. Please know that I am extremely grateful for your insightful remarks and important feedback. All of the criticisms that were made in my document have been handled, and I have finished the rewrite.

The abstract is clear but requires minor grammatical editing.

response to the reviewer

Thank you for your comment. We appreciate your positive assessment of the abstract. The abstract has been carefully revised and edited for grammar, clarity, and readability.

The questionnaire validation process needs more methodological detail (2-3 sentences).

Response to the reviewer

Thank you for this helpful comment. Additional details have been added to the Methods section to further clarify the questionnaire validation process.

The Data Availability statement (“available upon request”) may not meet PLOS policy (check upon re submission)

response to the reviewer

Thank you for highlighting this point. We updated the statement and thoroughly review the journal's data sharing policy upon resubmission to assure PLOS compliance.

Statistical methods are appropriate and clearly justified.

response to the reviewer

Thank you for your positive comment. We appreciate your assessment that the statistical methods were appropriate and clearly justified.

Add more specific policy recommendations in the conclusion section.

response to the reviewer

Thank you for this valuable suggestion, we have revised the conclusion section to include more specific. These now emphasise strengthening public awareness initiatives, improving physician-led vaccine counseling, expanding access to herpes zoster vaccination, and supporting national strategies that promote adult immunization uptake among high-risk populations

Reviewer #3:

I appreciate your consideration of my manuscript. I genuinely value your insightful criticism and considerate remarks. I have finalized the revision and responded to all suggestions in my text.

First, the manuscript requires comprehensive English language editing. Numerous grammatical and structural errors reduce clarity and hinder interpretation of the findings. Careful proofreading or professional language editing is strongly recommended.

Regarding the title, consistency with journal style should be considered, and sentence case formatting is recommended.

Response to the reviewer

Thank you for your comment. The title has been revised to follow sentence case formatting and to align with the journal’s style guidelines.

In the abstract, several statements require correction or clarification. The claim that 22.3% represents a majority of the population is inaccurate and should be revised. The Results section would benefit from clearer presentation of key outcomes. Additionally, the statement indicating no significant gender difference is incomplete and should specify the outcome variable being compared.

The description of the study population requires clarification. The methodology states that participants residing in Saudi Arabia were included; however, the results indicate that a proportion of participants were non-Saudi. The authors should clarify eligibility criteria and explain whether nationality or residency was the inclusion criterion. If residency was the basis for inclusion, this should be clearly stated. Furthermore, inconsistencies appear between the stated national scope of recruitment and references to recruitment from Riyadh. This discrepancy should be resolved.

Response to the reviewer

Thank you for this valuable comment. We appreciate the reviewer’s observation regarding the need to clarify the study population. We would like to clarify that the inclusion criterion was residency in Saudi Arabia rather than Saudi nationality. Therefore, both Saudi and non-Saudi individuals residing in Saudi Arabia were eligible for participation. The manuscript has been revised accordingly to clearly state this eligibility criterion and to ensure consistency regarding the national scope of recruitment throughout the manuscript.

The sample size calculation requires greater transparency. The parameters used in the Raosoft calculation, including assumed population proportion, confidence level, and margin of error, should be explicitly reported to allow reproducibility.

The questionnaire development process requires more detailed methodological description. The abstract indicates use of a bilingual questionnaire; however, translation procedures, languages used, and validation steps are not described in the methods section. The authors should clarify how translation was conducted and whether forward–backward translation or pilot testing was performed. Additionally, reliance on investigator input alone in questionnaire design may introduce measurement bias. Information on face validity, content validity, pilot testing, or reliability assessment should be provided. If the instrument was adapted from previous research, appropriate citation should be included. The use of online recruitment via social media platforms raises potential concerns regarding selection bias and geographic eligibility control; these issues should be acknowledged and discussed.

Response to the reviewer

Thank you for this valuable comment. We have revised the questionnaire development section to provide additional methodological details. Specifically, we clarified that the questionnaire was initially developed in English and translated into Arabic using forward and backward translation to ensure linguistic consistency. Furthermore, the questionnaire underwent pilot testing among 20 representative individuals to assess clarity, comprehension, and relevance before final distribution.

The statistical analysis section requires clarification regarding the derivation of the adverse event severity scale. The authors should specify whether severity categories were self-reported or based on predefined criteria. If the scale was adopted from prior literature, a reference should be provided; if developed for this study, justification and operational definitions are necessary.

Response to the reviewer

Thank you for this important comment. We have clarified the derivation of the adverse event severity measure in the revised manuscript. The severity variable was not adopted from a previously validated external scale. Rather, it was obtained from the researcher-developed questionnaire as a direct self-reported ordinal item for each vaccine dose. Participants rated the overall severity of their post-vaccination side effects using four predefined response categories: no symptoms, mild, moderate, and severe. Thus, the severity categories were self-reported by participants and were not assigned by clinicians. We also clarified in the Methods section that this variable reflected participant-perceived symptom severity and was analysed as an ordinal outcome.

In the results section, presentation should follow a consistent reporting format, such as N (%), throughout the manuscript. Statements referring to a “majority” should include corresponding percentages. Tables require clearer labeling and standardized formatting. Specifically, the classification of symptoms into mild, moderate, and severe requires explicit operational definitions and explanation of how these categories were assigned.

Response to the reviewer

Thank you for this helpful comment. We revised the Results section to ensure a more consistent reporting format throughout the manuscript. Specifically, descriptive findings are now presented more systematically using n (%) in both the text and tables, and statements such as “majority” or “most” were revised to include the corresponding frequencies and percentages. We also reorganized the descriptive presentation of the results by consolidating the original descriptive tables into three clearly labeled tables, which improved clarity and reduced overlap. In addition, subgroup-specific denominators are now explicitly identified, particularly for second-dose recipients (n = 152), in both the tables and their notes. Finally, the classification of symptom severity was clarified in the Methods section and table notes by explicitly stating that severity was measured as a self-reported ordinal item in the researcher-developed questionnaire using predefined categories of no symptoms, mild, moderate, and severe.

Attachment Submitted filename: Response to Reviewers.docx

# Peer review: Decision Letter 1

**DOI:** 10.1371/journal.pone.0360528.r003

**Authors:** Tungki Pratama Umar

20 Jul 2026

Dear Dr. ALDALI,

Thank you for submitting your manuscript to PLOS ONE. After careful consideration, we feel that it has merit but does not fully meet PLOS ONE’s publication criteria as it currently stands. Therefore, we invite you to submit a revised version of the manuscript that addresses the points raised during the review process.

==============================

ACADEMIC EDITOR COMMENTS (PLEASE SEE BELOW FOR DETAILS).

Please submit your revised manuscript by Sep 03 2026 11:59PM. If you will need more time than this to complete your revisions, please reply to this message or contact the journal office at plosone@plos.org. When you're ready to submit your revision, log on to https://www.editorialmanager.com/pone/ and select the 'Submissions Needing Revision' folder to locate your manuscript file.

- A letter that responds to each point raised by the academic editor and reviewer(s). You should upload this letter as a separate file labeled 'Response to Reviewers'.
- A marked-up copy of your manuscript that highlights changes made to the original version. You should upload this as a separate file labeled 'Revised Manuscript with Track Changes'.
- An unmarked version of your revised paper without tracked changes. You should upload this as a separate file labeled 'Manuscript'.

If you would like to make changes to your financial disclosure, please include your updated statement in your cover letter. Guidelines for resubmitting your figure files are available below the reviewer comments at the end of this letter.

If applicable, we recommend that you deposit your laboratory protocols in protocols.io to enhance the reproducibility of your results. Protocols.io assigns your protocol its own identifier (DOI) so that it can be cited independently in the future. For instructions see: https://journals.plos.org/plosone/s/submission-guidelines#loc-laboratory-protocols. Additionally, PLOS ONE offers an option for publishing peer-reviewed Lab Protocol articles, which describe protocols hosted on protocols.io. Read more information on sharing protocols at https://plos.org/protocols?utm_medium=editorial-email&utm_source=authorletters&utm_campaign=protocols.

As the corresponding author, your ORCID iD is verified in the submission system and will appear in the published article. PLOS supports the use of ORCID, and we encourage all coauthors to register for an ORCID iD and use it as well. Please encourage your coauthors to verify their ORCID iD within the submission system before final acceptance, as unverified ORCID iDs will not appear in the published article. *Only*  the individual author can complete the verification step; PLOS staff *cannot*  verify ORCID iDs on behalf of authors.

We look forward to receiving your revised manuscript.

Kind regards,

Tungki Pratama Umar, M.D., M.Sc.

Academic Editor

PLOS One

**Journal Requirements:**

If the reviewer comments include a recommendation to cite specific previously published works, please review and evaluate these publications to determine whether they are relevant and should be cited. There is no requirement to cite these works unless the editor has indicated otherwise.

**Additional Editor Comments:**

Thank you for your submission: Evaluating the Adverse Events Associated with Herpes Zoster Vaccination in Saudi Arabia: A Cross-Sectional Study. This is an interesting study. However, there are significant works that should be done to make it merit for publication. Here are my comments.

Introduction

1. Add more complications, such as meningoencephalitis and cardiovascular complications.

2. State whether similar publications exist in Saudi Arabia, and describe them along with your improvements.

3. This information is irrelevant because it does not assess HZV; a 2018 U.S. study highlighted serious adverse effects associated with varicella vaccination, including urticaria, ataxia, thrombocytopenia, pneumonia, encephalitis, stroke, and, in rare instances, death.

Methods

1. Study design: This is an overclaim (...in Saudi Arabia) since the study only assessed people in Riyadh, not all of the Saudi population. Please revise this information and the title.

2. Inclusion: …had been administered the herpes zoster vaccine (are patients with uncompleted schedules included, or is it strictly patients with completed vaccinations)?

3. Adverse event data were gathered using a well-organized questionnaire. Validation and reliability testing should be reported. The current validation technique is insufficient.

4. Power calculation for sample size should be reported alongside 95% CI.

5. Please state sampling methodology (random or non-random). Are there any further inclusion and exclusion criteria?

6. The pilot interview includes 20 individuals representative of the target study population. How can the authors ensure about the sample's representativeness? Gender, education, profile, etc? Please describe more.

7. Please attach the questionnaire given to participants as supplementary material (including the instructions).

8. Is there any standard on how the adverse events are assessed? Mild, moderate, and severe definitions should be described thoroughly.

9. ...compare severity scores across multiple age groups. Please state the source of how patients are clustered based on age group.

10. Add post-hoc analysis for Kruskal-Wallis.

Results

1. The authors just repeat information in the table, which is an unfavorable practice. Please summarize the information from... As shown in Table 1, the gender distribution was nearly equal, with 193 females (50.5%) and 189 males (49.5%).

2. Table 2 should be analyzed statistically since some parameters such as feeling tired (revise to fatigue), headache, temperature or chills (please revise this classification since it is vague), and no adverse events may have differences (based on percentage).

3. The data in Tables 2 and 3 do not match regarding adverse events (please recheck since it is confusing and just a repetition). Furthermore, tables 2 and 3 stated relatively similar topics that are complementary to each other. Please merge Tables 2 and 3, and include a statistical analysis as well.

1. Table 2: No adverse effects: 112 (29.3%), 28 (18.4%)

2. Table 3: No symptoms: 115 (30.1%) and 28 (18.4%)

3. Table 2 onset No symptoms 121 (31.7%) 30 (19.7%)

4. Table 3 duration: No symptoms 116 (30.4%) 30 (19.7%)

4. Add types of statistical analysis below each table.

5. … using the Mann–Whitney U test and the Kruskal–Wallis test. To provide a more informative interpretation of the results, effect sizes were dividing it into two groups and analyzing it for multivariate regression alongcalculated for the non-parametric comparisons. For Mann–Whitney U tests, effect size r was computed as |Z|/√N, and for the Kruskal–Wallis test, epsilon-squared (ε²) was reported. (Not important in the result; can be added in the methods)

6. Table 4 -> assess composite data also (regardless of dose or smoking status).

7. Do the analysis on the total number of adverse events, regardless of doses (in addition to currently available data).

8. For p-value, under the table, add "*" for significant results, and state the threshold and statistical tests. Do not add statistical tests in the table title.

9. Tables 4 to 7 can be merged and interpreted simultaneously.

10. A multivariate analysis (logistic regression) is highly recommended to determine which factors are the most significant contributors while highlighting the confounders.

11. Where is the post-hoc information on table 8?

12. For age, consider also dividing it into two groups and analyzing it for multivariate regression also with other parameters (tables 4-7). Search for the most appropriate classification (such as >=50 or <50) based on the available literature.

DISCUSSION

1. The second paragraph should be divided into two or three paragraphs given its long form. Also, there is inadequate citation on it, particularly in the first 15+ lines. Please add more citations to any part of the statement or claim.

2. Inconsistent citation style: Similar gaps in vaccine literacy have been documented in prior studies such as Hurley et al. (2018).

3. Following multivariate regression, revise this section. State that from univariate or bivariate perspectives, they can have a significant result. However, in multivariate analyses, certain interactions may be eliminated.

4. Why are most participants aged 18–40 years? The CDC guideline mostly targets this vaccination for people aged >50.

5. Sampling may be flawed. How can the authors arrive at 382 samples in the final analysis? People who filled out the forms are included, as this is very commonly done.

CONCLUSION

1. The conclusion is very stretched. Please focus on the main objective and do not describe too much of the study procedure. State several main results, particularly from regression statistics.

GENERAL

1. Some parts of the manuscript look refined or created through AI assistance. Please declare it as a statement of AI DISCLOSURE AND DECLARATION (please check PLoS One guideline).

2. Check on English proofreading comment

[Note: HTML markup is below. Please do not edit.]

Reviewers' comments:

Reviewer's Responses to Questions

**Comments to the Author**

Reviewer #2: All comments have been addressed

Reviewer #4: All comments have been addressed

**********

2. Is the manuscript technically sound, and do the data support the conclusions?

Reviewer #2: Yes

Reviewer #4: Yes

**********

3. Has the statistical analysis been performed appropriately and rigorously?

Reviewer #2: Yes

Reviewer #4: Yes

**********

4. Have the authors made all data underlying the findings in their manuscript fully available?

The PLOS Data policy

Reviewer #2: Yes

Reviewer #4: Yes

**********

5. Is the manuscript presented in an intelligible fashion and written in standard English?

Reviewer #2: Yes

Reviewer #4: Yes

**********

Reviewer #2: I have some suggestions:

1. English proofreading is required.

2. The high percentage of participants who were unable to identify the vaccine type may affect the validity of comparisons between Zostavax and Shingrix. (Add this to the limitatuion section).

No other comments

Reviewer #4: (No Response)

**********

what does this mean?). If published, this will include your full peer review and any attached files.

If you choose “no”, your identity will remain anonymous but your review may still be made public.

**Do you want your identity to be public for this peer review?**  For information about this choice, including consent withdrawal, please see our Privacy Policy

Reviewer #2: **Yes:**  Nasser M Alorfi

Reviewer #4: **Yes:**  Rehab H. Werida, Ph.D - Faculty of Pharmacy, Damanhour University, Egypt.

**********

[NOTE: If reviewer comments were submitted as an attachment file, they will be attached to this email and accessible via the submission site. Please log into your account, locate the manuscript record, and check for the action link "View Attachments". If this link does not appear, there are no attachment files.]

To ensure your figures meet our technical requirements, please review our figure guidelines: https://journals.plos.org/plosone/s/figures

You may also use PLOS’s free figure tool, NAAS, to help you prepare publication quality figures: https://journals.plos.org/plosone/s/figures#loc-tools-for-figure-preparation.

NAAS will assess whether your figures meet our technical requirements by comparing each figure against our figure specifications.

# Author comment: Author response to Decision Letter 2

**DOI:** 10.1371/journal.pone.0360528.r004

14 Sep 2026

We sincerely thank you for your careful evaluation of our manuscript and for your constructive comments and valuable suggestions. Your feedback has helped us improve the manuscript’s clarity, methodological presentation, and overall scientific quality. We have carefully addressed all comments and incorporated the corresponding revisions into the manuscript. We hope that the revised version is now suitable for publication.

Attachment Submitted filename: Response to Reviewers 2-9-2026.docx

# Editor report: Decision Letter 2

**DOI:** 10.1371/journal.pone.0360528.r005

**Authors:** Tungki Pratama Umar

29 Sep 2026

Adverse Events of Herpes Zoster Vaccination Among Adults in Saudi Arabia: A Cross-Sectional Study

PONE-D-25-51374R2

Dear Dr. ALDALI,

We’re pleased to inform you that your manuscript has been judged scientifically suitable for publication and will be formally accepted for publication once it meets all outstanding technical requirements.

Within one week, you’ll receive an e-mail detailing the required amendments. When these have been addressed, you’ll receive a formal acceptance letter and your manuscript will be scheduled for publication.

An invoice will be generated when your article is formally accepted. Please note, if your institution has a publishing partnership with PLOS and your article meets the relevant criteria, all or part of your publication costs will be covered. Please make sure your user information is up-to-date by logging into Editorial Manager at Editorial Manager® and clicking the ‘Update My Information' link at the top of the page. For questions related to billing, please contact billing support.

If your institution or institutions have a press office, please notify them about your upcoming paper to help maximize its impact. If they’ll be preparing press materials, please inform our press team as soon as possible -- no later than 48 hours after receiving the formal acceptance. Your manuscript will remain under strict press embargo until 2 pm Eastern Time on the date of publication. For more information, please contact onepress@plos.org.

Kind regards,

Tungki Pratama Umar, M.D.

Academic Editor

PLOS One

Additional Editor Comments (optional):

Reviewers' comments:

# Editor report: Acceptance letter

**DOI:** 10.1371/journal.pone.0360528.r006

**Authors:** Tungki Pratama Umar

PONE-D-25-51374R2

PLOS One

Dear Dr. ALDALI,

I'm pleased to inform you that your manuscript has been deemed suitable for publication in PLOS One. Congratulations! Your manuscript is now being handed over to our production team.

At this stage, our production department will prepare your paper for publication. This includes ensuring the following:

* All references, tables, and figures are properly cited

* All relevant supporting information is included in the manuscript submission,

* There are no issues that prevent the paper from being properly typeset

You will receive further instructions from the production team, including instructions on how to review your proof when it is ready. Please keep in mind that we are working through a large volume of accepted articles, so please give us a few days to review your paper and let you know the next and final steps.

Lastly, if your institution or institutions have a press office, please let them know about your upcoming paper now to help maximize its impact. If they'll be preparing press materials, please inform our press team within the next 48 hours. Your manuscript will remain under strict press embargo until 2 pm Eastern Time on the date of publication. For more information, please contact onepress@plos.org.

You will receive an invoice from PLOS for your publication fee after your manuscript has reached the completed accept phase. If you receive an email requesting payment before acceptance or for any other service, this may be a phishing scheme. Learn how to identify phishing emails and protect your accounts at https://explore.plos.org/phishing.

If we can help with anything else, please email us at customercare@plos.org.

Thank you for submitting your work to PLOS One and supporting open access.

Kind regards,

PLOS One Editorial Office Staff

on behalf of

Dr. Tungki Pratama Umar

Academic Editor

PLOS One
